# USP9X as a Candidate Mediator of Prenatal Aspirin‐Induced Ovarian Reserve Reduction in Offspring Mice

**DOI:** 10.1002/advs.202507679

**Published:** 2026-01-12

**Authors:** Yating Li, Caiyun Ge, Wai Yen Yim, Hui Feng, Tiancheng Wu, Lu Chen, Qiaohua Xiong, Shumin Pan, Mei Wang, Huijun Chen, Yuanzhen Zhang, Hui Wang

**Affiliations:** ^1^ Department of Obstetrics and Gynaecology Zhongnan Hospital of Wuhan University Wuhan China; ^2^ Department of Pharmacology Basic Medical School of Wuhan University Wuhan China; ^3^ Hubei Provincial Key Laboratory of Developmentally Originated Disease Wuhan China; ^4^ Reproductive Medicine Center Zhongnan Hospital of Wuhan University Wuhan China; ^5^ Department of Cardiovascular Surgery Union Hospital Tongji Medical College Huazhong University of Science and Technology Wuhan Hubei China; ^6^ Clinical Research Center for Prenatal Diagnosis and Birth Health of Hubei Province Wuhan China; ^7^ Clinical Research Center for Reproductive Science and Birth Health of Wuhan Wuhan China

**Keywords:** diminished ovarian reserve, prenatal aspirin exposure, primordial follicle assembly, ubiquitin‐specific protease 9X (USP9X)

## Abstract

Aspirin, widely used during pregnancy to prevent complications, may adversely affect fetal development. This study investigates prenatal aspirin exposure (PAE) on ovarian reserve in female offspring. Pregnant mice received aspirin (5, 10, and 20 mg/kg·d) from gestational days 918. Morphological and functional analyses of ovaries across prenatal to postnatal stages revealed PAE (20 mg/kg·d) reduced primordial and antral follicle counts, increased follicular atresia, and impaired ovulation in adulthood. Transcriptomics identified sustained downregulation of *Usp9x* as a potential mediator, which correlated with suppression of the HIF1α/NOBOX signaling axis. Mechanistically, our data suggest that aspirin exposure is associated with increased HDAC1 activity and enrichment of HDAC1 at the *Usp9x* promoter, accompanied by a reduction of H3K27ac marks, thereby potentially epigenetically silencing *Usp9x* expression. This enhanced HIF1α ubiquitination and degradation, ultimately attenuating NOBOX‐mediated transcriptional regulation of downstream follicular genes. Using in vitro fetal ovarian cultures and NIH3T3 cells, we confirmed aspirin's disruption of primordial follicle assembly via the USP9X‐HIF1α‐NOBOX pathway. Crucially, HDAC1 knockdown or pharmacological inhibition rescued USP9X expression and restored follicular development. Our findings indicate that PAE reduces ovarian reserve through HDAC1‐linked epigenetic silencing of *Usp9x*, exacerbating HIF1α/NOBOX pathway dysfunction, thereby informing aspirin's gestational safety and future interventions for fetal‐origin ovarian disorders.

## Graphical abstract

This study suggests that prenatal aspirin exposure is associated with reduced ovarian reserve in offspring, associated with HDAC1‐linked epigenetic downregulation of *Usp9x* as a candidate mechanism. These preclinical findings provide new insights into fetal‐origin ovarian disorders and contribute to the evidence base concerning aspirin's gestational safety.

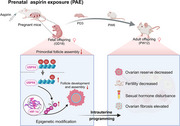

## 1 Introduction

Aspirin, a nonsteroidal anti‐inflammatory drug (NSAID), is increasingly utilized during pregnancy not only for analgesia and anti‐inflammation but also for preventing preeclampsia, recurrent miscarriage, intrauterine growth restriction, and vasculopathic fetal death [1]. Epidemiological surveillance reveals a progressive rise in prenatal aspirin use, with utilization rates reaching 2%–5% in Western countries and up to 14% in Middle Eastern regions [2]. Classified as a U.S. Food and Drug Administration (FDA) Pregnancy Category C medication, aspirin readily crosses the placental barrier with fetal clearance capacity substantially lower than adult metabolic rates [3]. Clinical evidence indicates that prenatal aspirin exposure (PAE) increases risks of fetal intracranial hemorrhage, neural tube defects, testicular endocrine disruption, cleft palate, gastroschisis, and renal impairment [4, 5, 6, 7]. These effects may persist postnatally, elevating susceptibility to offspring asthma, spastic cerebral palsy, and schizophrenia. Although studies demonstrate aspirin's detrimental effects on parental ovarian development, including in vivo and in vitro suppression of folliculogenesis, ovulation, and cumulus granulosa cell estrogen synthesis [8, 9], its impact on offspring ovarian development and adult reproductive function remains unexplored.

Ovarian reserve, defined as the quantity and quality of residual follicles within the ovaries, serves as a critical biomarker of female reproductive potential. Diminished ovarian reserve (DOR), characterized by reduced oocyte quantity and/or quality, constitutes a significant public health concern due to its detrimental effects on fertility and transgenerational health outcomes [10, 11, 12]. Despite rising DOR incidence rates, its pathogenesis remains poorly understood, hampering early intervention strategies. Emerging evidence suggests maternal gestational exposures (e.g., acetaminophen, azithromycin, and high‐fat diet) can impair offspring ovarian reserve and steroidogenic capacity [13, 14, 15, 16]. Notably, primordial follicle assembly disruption has been identified as a potential mechanism underlying fetal‐origin DOR [17]. This developmental process, involving oocyte nest breakdown and subsequent encapsulation of individual oocytes by flattened granulosa cells, primarily occurs during late gestation and early postnatal stages [18]. As a critical window of ovarian developmental sensitivity, primordial follicle assembly exhibits particular vulnerability to environmental insults [19]. Whether PAE affects this fundamental process remains unknown.

In this investigation, we established PAE mice to systematically evaluate PAE‐induced ovarian morphological and functional alterations throughout prenatal and postnatal development. Through integrated transcriptomic profiling, in vitro fetal ovarian culture, and cellular intervention approaches, we explore the potential mechanisms underlying PAE‐induced ovarian dysfunction. This study aims to establish a preclinical model and elucidate a potential mechanism underlying PAE‐induced ovarian dysfunction, which could, in the future, contribute to the foundation for evaluating risks and designing protective strategies.

## 2 Results

### 2.1 PAE Leads to Reduced Ovarian Reserve in Adult Female Offspring Mice

**FIGURE 1:**
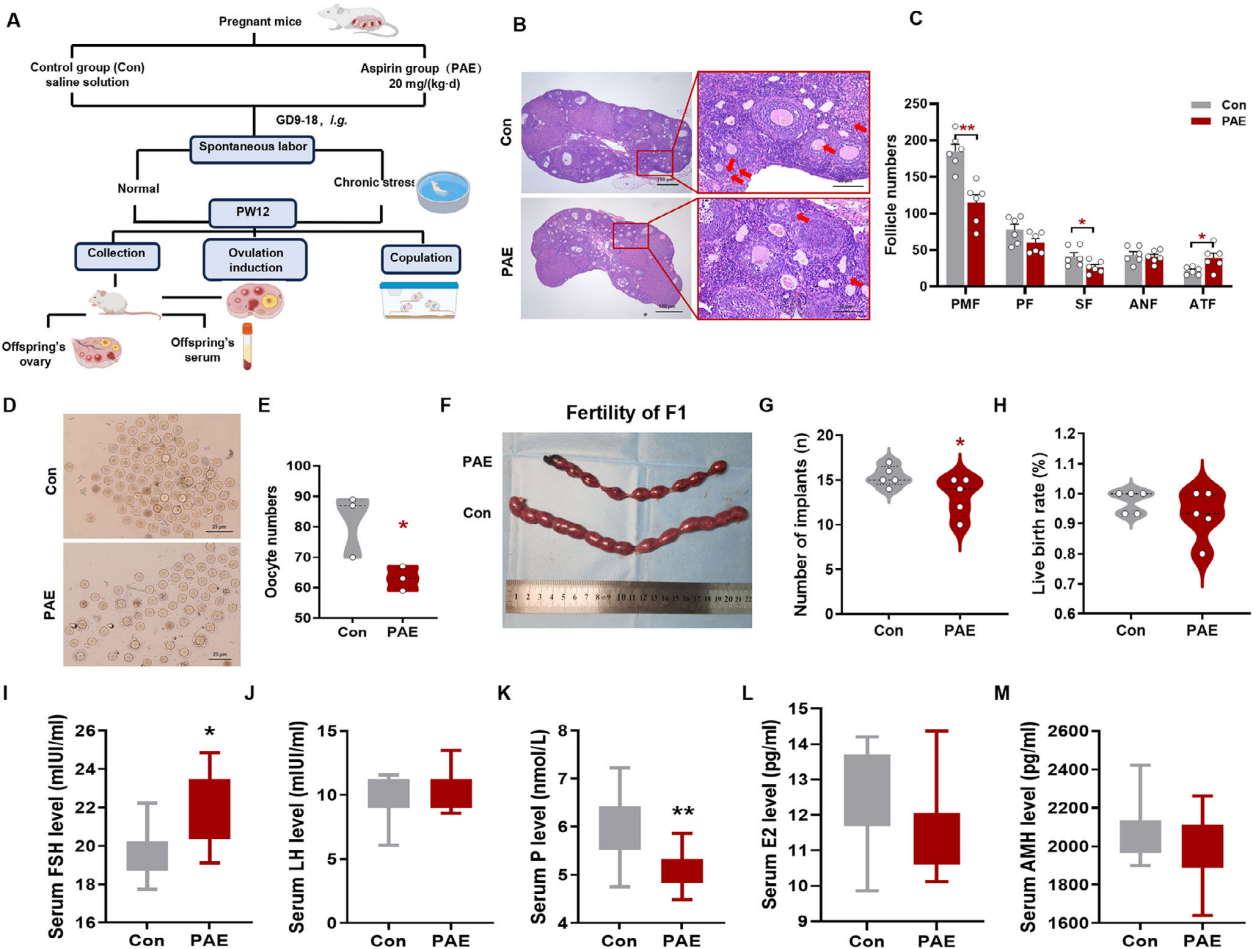
Effects of PAE on ovarian morphology and function in female offspring mice at PW12. (A) Schematic diagram of animal conduct; (B) Ovarian morphology (H&E), *n* = 5; (C) Number of follicles, *n* = 5; (D) Ovulation stimulating oocyte image, *n* = 3; (E) Number of oocytes after ovulation, *n* = 5; (F) Image of embryo implantation in the uterus offspring after mating; (G) Number of implants, *n* = 5; (H) Live birth rate, *n* = 5; (I–M) Serum E2, P, FSH, LH, and AMH concentrations, *n* = 10. Mean ± S.E.M. ^*^ *p <* 0.05, ^**^ *p* < 0.01 vs. control. PAE, prenatal aspirin exposure; PW, postnatal week; E2, estradiol; P, progesterone, FSH, follicle‐stimulating hormone; LH, luteinizing hormone; AMH, anti‐Müllerian hormone; PMF, primordial follicle; PF, primary follicle; SF, secondary follicle; ANF, antral follicle; ATF, atretic follicles.

To investigate the effects of prenatal aspirin exposure (PAE) on ovarian development and reproductive function, we established a mouse model in which pregnant mice were administered aspirin at 20 mg/kg·d from gestational day (GD) 9 to 18. This dose was selected to reflect clinically relevant exposure, based on human low‐dose aspirin regimens (100–162 mg/d) recommended for preeclampsia prevention in high‐risk pregnancies. The mouse equivalent dose was calculated using the FDA‐recommended body surface area (BSA) normalization method for interspecies dose conversion, which yielded 20.56 mg/kg·d from a typical human dose of 100 mg per 60 kg individual [20]. As illustrated in Figure 1A, we first analyzed ovarian morphology and functional outcomes in adult female offspring at postnatal week 12 (PW12). Compared to controls, PAE mice exhibited significant reductions in primordial and secondary follicle counts (Figure 1B,C) and increased atretic follicles. Superovulation assays revealed significantly fewer oocytes in PAE mice (Figure 1D,E). Mating trials demonstrated decreased embryo implantation and live birth rates in the PAE group (Figure 1F–H). Similar reductions in primordial and antral follicles were observed at PW6 (Figure S1A,B). To assess the long‐term impact of PAE on ovarian resilience, adult offspring were subjected to chronic stress from PW10 to PW12. The number of follicles at all stages was found to be generally reduced in PW12‐stress (Figure S1C,D). Follicle quantification revealed a differential response between groups. In Control mice, stress significantly reduced primordial and antral follicle counts. In contrast, PAE‐group ovaries exhibited a distinct pathological response: while stress did not further deplete primordial or mature follicles beyond the baseline PAE deficit, it significantly increased follicular atresia (Figure S1E). This indicates that PAE altered the ovarian stress response, leading to accelerated follicle degeneration rather than uniform depletion. PAE offspring showed marked downregulation of folliculogenesis‐related genes (*Nobox*, *Sohlh1*, *Gdf9*) at PW12, with further suppression of *Figlα*, *Sohlh1*, *Gdf9*, *Bmp15*, *Kit*, and *Kitl* under stress (Figure S1F,G), confirming DOR. Endocrine profiling revealed elevated follicle‐stimulating hormone (FSH) (Figure 1I) and reduced progesterone levels (Figure 1K), while estrogen, luteinizing hormone (LH), and anti‐Müllerian hormone (AMH) remained unchanged (Figure 1J,L,M). Transcriptomic analysis identified 604 differentially expressed genes (337 upregulated, 267 downregulated) in PAE ovaries. KEGG pathway enrichment highlighted suppressed steroid hormone biosynthesis, corroborated by downregulated mRNA expression of steroidogenic enzymes (*Star*, *Cyp11a1*, *Cyp19a1*, *3β‐Hsd*, *17β‐Hsd1*) (Figure S2). Collectively, these findings demonstrate that PAE impairs ovarian reserve, fertility, and steroidogenic capacity in adult female offspring.

### 2.2 Decreased Ovarian Reserve Function in Female Offspring Mice of PAE Mice is Associated With Abnormal Follicular Development and Assembly

**FIGURE 2:**
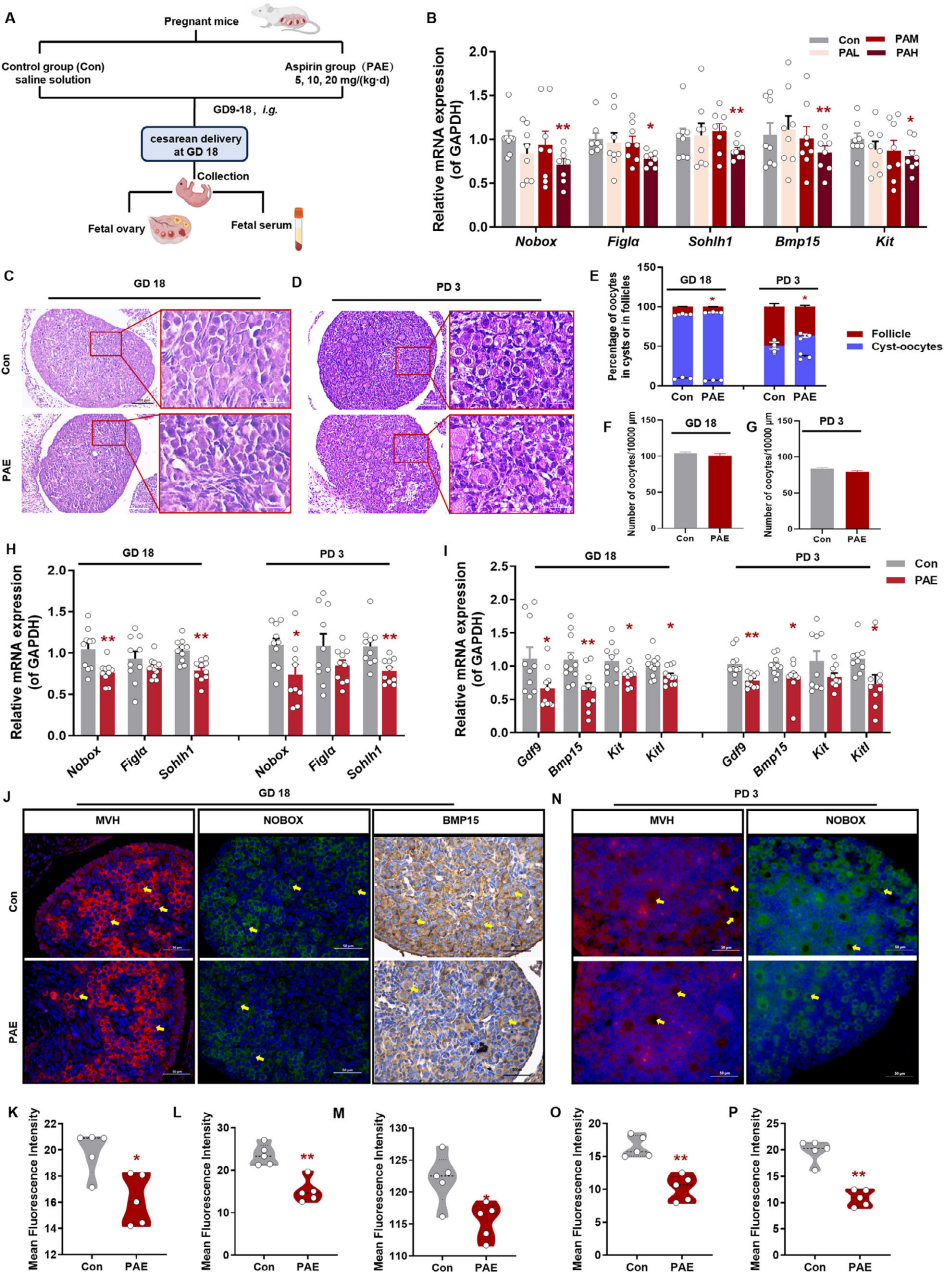
Effects of PAE on ovarian morphology and function in female offspring mice at GD18 and PD3. (A) Schematic diagram of animal conduct; (B) Relative mRNA expression of genes related to oocyte development and primordial follicle assembly of ovaries under different concentrations of aspirin, *n* = 10; (C, D) Ovarian morphology by H&E staining, *n* = 5; (E) Total oocyte number of fetal ovary, *n* = 5; (F) Total oocyte number of PD3 offspring ovary, *n* = 5; (G) The percentage of oocytes in cysts, *n* = 5; (H) The relative mRNA expression of *Nobox, Figlα*, and *Sohlh1*, *n* = 10; (I) The relative mRNA expression of *Gdf9, Bmp15, Kit*, and *Kitl*, *n* = 10; (J) IF and IHC staining of MVH, NOBOX and BMP15 (the MVH positive cells stained by red, the NOBOX positive cells stained by green, the oocyte indicated by yellow arrow), *n* = 5; (K‐M) The relative mean fluorescence intensity of MVH, NOBOX, and BMP15; (N) IF staining of MVH and NOBOX, *n* = 5; (O, P) The relative mean fluorescence intensity of MVH and NOBOX. Mean ± S.E.M. ^*^ *p* < 0.05, ^**^ *p* < 0.01 vs. control. PAE, prenatal aspirin exposure; GD, gestational day; PD, postnatal day; *Nobox*, newborn ovary homeobox gene; *Figlα*, factor in the germline alpha; *Sohlh1*, spermatogenesis and oogenesis specific basic helix‐loop‐helix 1; *Gdf9*, growth differentiation factor 9; *Bmp15*, bone morphogenetic protein 15; *Kit*, receptor tyrosine kinase; *Kitl*, kit ligand; PAE(L), low concentration prenatal aspirin exposure; PAE(M), medium concentration prenatal aspirin exposure; PAE(H), high concentration prenatal aspirin exposure; IF, immunofluorescence; IHC, immunohistochemistry.

To investigate the impact of PAE on primordial follicle assembly before and after birth, we treated pregnant mice with different doses (5, 10, and 20 mg/kg·d) of aspirin (Figure 2A). Key regulators of primordial follicle assembly, including oocyte‐specific transcription factors (*Nobox*, *Figlα*, *Sohlh1*) and critical signaling components (*Gdf9*, *Bmp15*, *Kit*, *Kitl*) [21] were assessed. High‐dose PAE (20 mg/kg·d) significantly reduced mRNA expression of oocyte development‐related genes in fetal ovaries (Figure 2B). Spearman correlation analysis indicated that the expression levels of the pivotal oogenic transcription factors *Nobox* (R = −0.38, *p* = 0.034) and *Figlα* (R = −0.47, *p* = 0.0072) showed a negative correlation with increasing doses of prenatal aspirin exposure (Figure S3). Given that the most pronounced and consistent phenotypic disruption in primordial follicle assembly was observed at the 20 mg/kg·d dose, we selected this high‐dose exposure model for all subsequent experiments. H&E staining revealed morphological abnormalities in PAE‐exposed GD18 and PD3 ovaries, characterized by disorganized germ cell nests and pyknotic nuclei in cortical oocytes (Figure 2C,D). We observed a significant reduction in the ratio of oocytes within follicles to total oocytes (Figure 2E), despite no significant difference in the total oocyte count between PAE and control groups at both GD18 and PD3 (Figure 2F,G). These findings suggest that the impairment in primordial follicle assembly, rather than a depletion of the primordial oocyte pool, underlies the subsequent decline in ovarian reserve.

PAE suppressed mRNA expression of follicular assembly regulators across developmental stages, with significant reductions in *Nobox* and *Sohlh1* as well as *Bmp15*, *Gdf9*, *Kit*, and *Kitl* (Figure 2H,I). Immunofluorescence and immunohistochemistry confirmed decreased protein levels of oocyte‐specific markers MVH, NOBOX, and BMP15 in PAE offspring (Figure 2J–P). These findings collectively demonstrate that PAE disrupts follicular development and primordial follicle assembly in female offspring during critical prenatal and postnatal stages.

### 2.3 USP9X is Associated With Abnormal Primordial Follicle Development and Assembly in Female Offspring of PAE Mice

**FIGURE 3:**
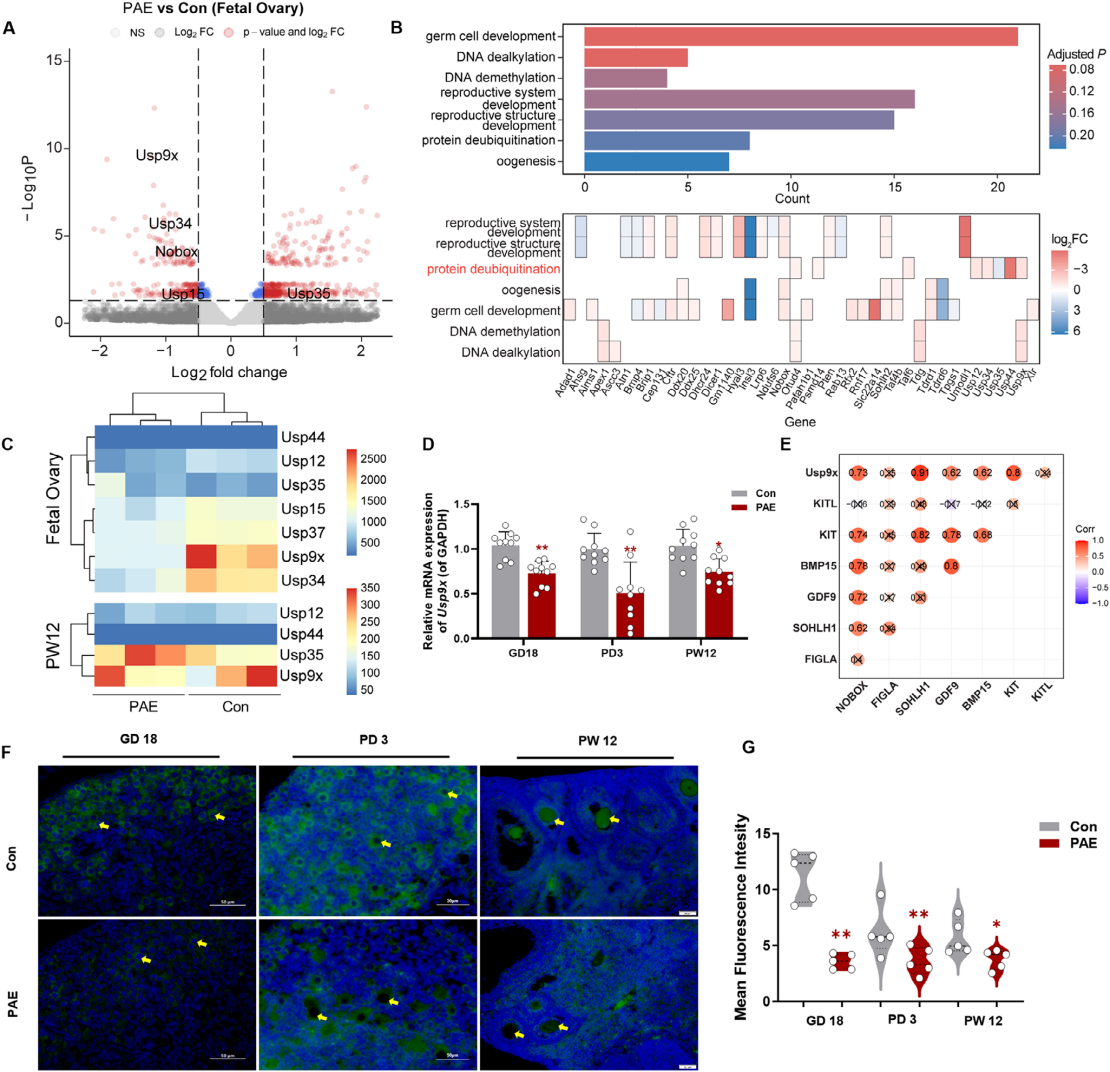
The change of USP9X in offspring mice ovaries. (A) Volcano plot showing the differential expressed genes in PAE fetal ovary compared with controls, *n* = 3; (B) Barplot showing enriched Reactome pathways using differentially expressed genes in fetal ovaries; (C) Reactome pathway enrichment analysis of differentially expressed genes from RNA sequencing results in fetal and PW12 offspring ovaries; (D) The relative mRNA expression of *Usp9x* in the ovaries of PAE offspring at GD18, PD3, and PW12, *n* = 10; (E) Heat map analysis of mRNA relative expression levels of *Usp9x* and *Nobox, Figlα, Sohlh1, Bmp15, Gdf9, Kit* and *Kitl* in fetal ovaries; (F) IF staining of USP9X, USP9X positive cells stained by green, the oocyte indicated by yellow arrow, *n* = 5; (G) The mean fluorescence intensity of USP9X protein expression by IF, *n* = 5. Mean ± S.E.M. ^*^p< 0.05, ^**^p< 0.01 vs. control. USP9X, ubiquitin specific protease 9, X‐linked; PAE, prenatal aspirin exposure; *Nobox*, newborn ovary homeobox gene; *Figlα*, factor in the germline alpha; *Sohlh1*, spermatogenesis and oogenesis specific basic helix‐loop‐helix 1; *Gdf9*, growth differentiation factor 9; *Bmp15*, bone morphogenetic protein 15; *Kit*, receptor tyrosine kinase; *Kitl*, kit ligand; IF, immunofluorescence; GD, gestational day; PD, postnatal day; PW, postnatal week.

To explore the mechanism by which aspirin affects primordial follicle development and assembly, we performed RNA sequencing and analysis on fetal ovarian tissues. Compared to the control group, we identified 155 differentially expressed genes in the PAE group, with 103 upregulated and 52 downregulated (Table S1). *Usp9x* expression was severely repressed following PAE (PAE TPM: 942.2), placing it among the Top 3 most downregulated genes in the entire dataset (Figure 3A). Reactome pathway enrichment analysis revealed significant suppression of protein deubiquitination pathways (Figure 3B). To pinpoint the specific deubiquitinating enzyme (DUB) responsible for mediating PAE‐induced pathology, we performed a meticulous analysis of the DUBs identified in the suppressed protein deubiquitination pathway. The level of *Usp12, Usp34, Usp35, Usp44*, and *Usp9x* decreased, with the decrease in *Usp9x* being the most significant (TPM in PAE vs Con: 942.2 vs 2208.8), and in the ovarian RNA‐seq of PW12, *Usp9x* was the deubiquitinating enzyme with the highest expression level (Figure 3C). RT‐qPCR analysis confirmed that *Usp9x* mRNA expression was downregulated in the ovaries of PAE offspring at GD18, PD3, and PW12 (Figure 3D). In contrast, the expression levels of *Usp34* and *Usp37* mRNA in GD18 PAE offspring ovaries remained unchanged (Figure S4A,B). Correlation analysis revealed that *Usp9x* expression showed a strong positive correlation with established critical oogenic transcription factors, including *Sohlh1* (R = 0.91), *Nobox* (R = 0.73), *Gdf9* (R = 0.62), *Bmp15* (R = 0.62), and *Kit* (R = 0.8) (Figure 3E; Figure S4C–G). Immunofluorescence results further showed a significant decrease in USP9X protein levels (Figure 3F,G). These results indicate that PAE‐induced USP9X downregulation is associated with and may contribute to follicular developmental defects.

**FIGURE 4:**
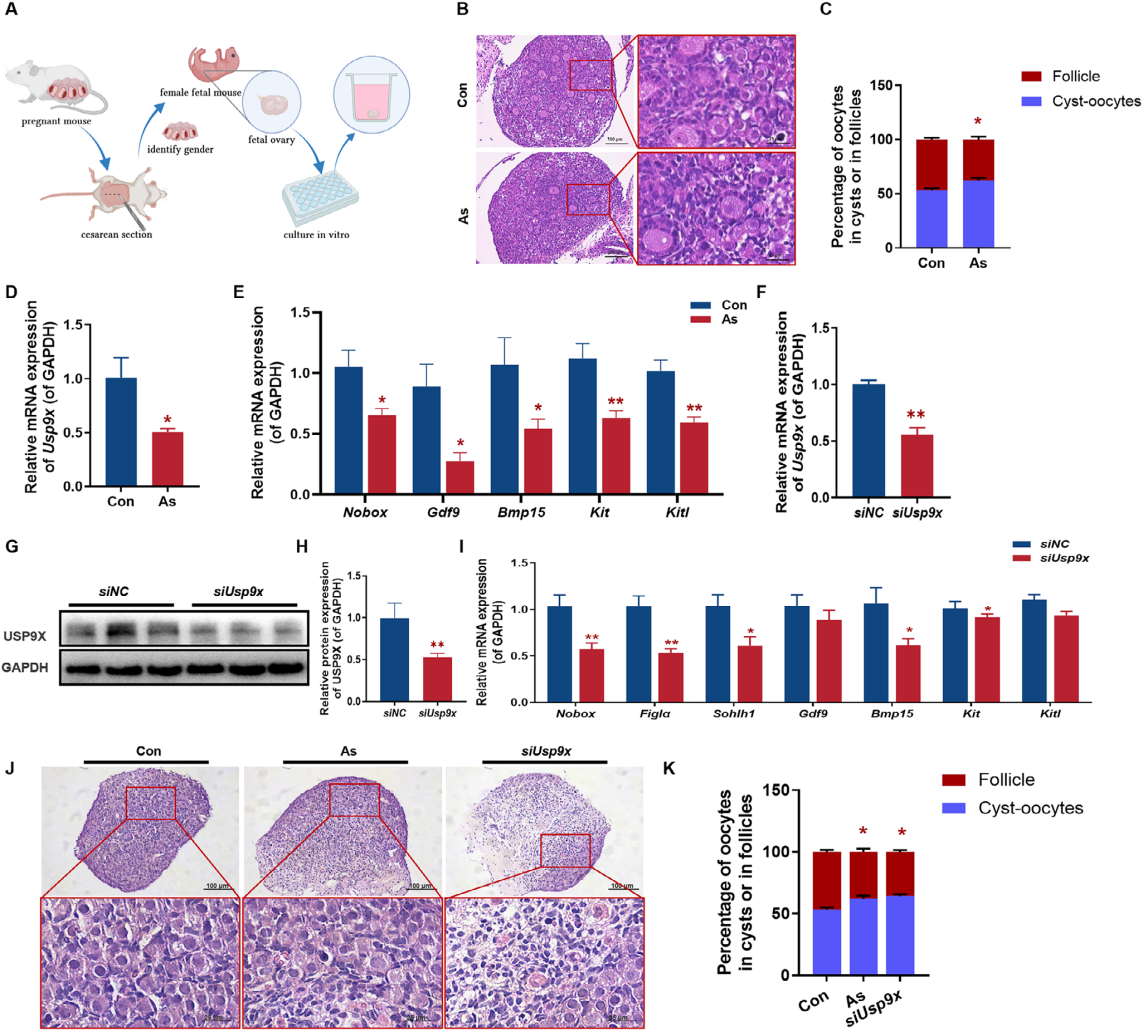
The impact of USP9X knockout on in vitro cultured fetal ovaries. (A) Schematic diagram of fetal ovary culture model construction in vitro; (B) H&E staining in fetal ovaries in vitro, *n* = 5; (C) The percentage of oocytes in cysts, *n* = 5; (D) The relative mRNA expression of *Usp9x*, *n* = 10; (E) The relative mRNA expression of follicle development gene, *n* = 10; (F) The relative mRNA expression of *Usp9x*, *n* = 10; (G) Protein expression of USP9X, *n* = 5; (H) The relative protein expression of USP9X, *n* = 5; (I) The relative mRNA expression of follicle development gene, *n* = 10; (J) H&E staining of usp9x‐knockdown ovaries in vitro, *n* = 5; (K) The percentage of in cysts of in follicles, *n* = 5. Mean ± S.E.M. ^*^p< 0.05, ^**^p< 0.01 vs. control. USP9X, ubiquitin‐specific protease 9, X‐linked; Nobox, newborn ovary homeobox gene; Figlα, factor in the germline alpha; Sohlh1, spermatogenesis and oogenesis specific basic helix‐loop‐helix 1; Gdf9, growth differentiation factor 9; Bmp15, bone morphogenetic protein 15; Kit, receptor tyrosine kinase; Kitl, kit ligand; GD, gestational day.

Ovary culture in vitro is a useful model for studying follicular and oocyte development damage and helps elucidate the mechanisms of ovarian development. We established an in vitro culture system for fetal ovaries from GD18 with varying aspirin concentrations (1, 10, and 100 µm) to explore the mechanism underlying the decreased ovarian reserve function induced by PAE (Figure 4A). This concentration aligns with pharmacokinetic data from human studies, in which reported peak plasma levels of salicylic acid, the active metabolite of aspirin, in pregnant women receiving low‐dose regimens (100–150 mg/d) range between approximately 17.5 and 23.8 µm [22]. Furthermore, our preliminary dose‐response assay identified 10 µm as the most effective concentration that suppressed *Usp9x* and oocyte development‐related genes (*Nobox*, *Figlα*, *Gdf9*, *Bmp15*) (Figure S4H,I). Therefore, fetal ovaries were treated with 10 µm aspirin in all subsequent experiments. H&E staining showed that aspirin treatment led to disordered oocyte nests and cords in the cultured fetal ovaries (Figure 4B), and a reduced ratio of follicular oocytes to total oocytes (Figure 4C). Moreover, the mRNA expression of *Usp9x* and primordial follicle assembly‐related genes, such as *Nobox, Gdf9*, *Bmp15*, *Kit*, and *Kitl*, were significantly reduced in the aspirin‐treated group (Figure 4D,E), confirming aspirin's inhibitory effect on primordial follicle assembly.

Although USP9X is known to regulate oocyte and follicular development, its role in primordial follicle assembly remains unexplored. To address this, we performed USP9X knockdown using siRNA in cultured fetal ovaries. The siUsp9x group exhibited significant reductions in *Usp9x* mRNA and protein levels (Figure 4F–H). This knockdown led to suppressed expression of oogenic transcription factors (*Nobox*, *Figlα*, *Sohlh1*) (Figure 4I). The morphological analysis further confirmed the functional consequence of USP9X downregulation. Consistent with PAE treatment, *siUsp9x* significantly impaired primordial follicle formation, resulting in a dramatic reduction in the ratio of follicular oocytes to total oocytes (Figure 4J,K). This finding demonstrates that perturbation of USP9X can replicate the core molecular and morphological defects of PAE during primordial follicle assembly, highlighting its non‐redundant role in this process.

### 2.4 Ubiquitination of HIF1α Enhances the Downregulation of Follicular Development‐Related Gene Expression Associated With USP9X

**FIGURE 5:**
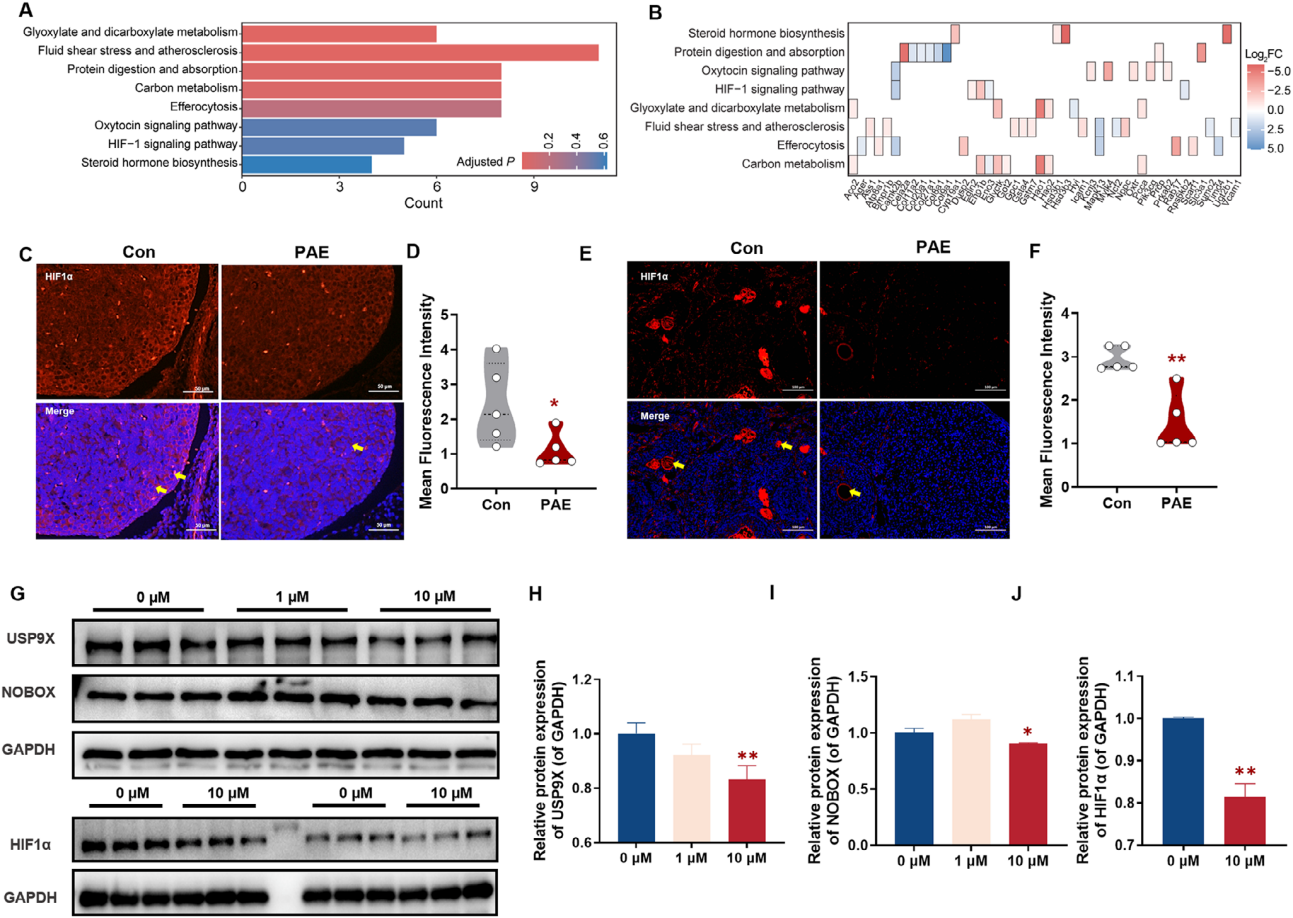
Changes of USP9X‐Hif1α‐NOBOX pathway in offspring mice ovaries. (A) Bar plot displaying enriched KEGG pathways identified from differentially expressed genes in ovaries of PW12 offspring, *n* = 3; (B) Heatmap illustrating the expression patterns of genes within the enriched KEGG pathways; (C,E) IF staining images of HIF1α in GD18 and PW12 offspring ovaries, the oocyte indicated by yellow arrow, *n* = 5; (D, F) Relative mean fluorescence intensity of HIF1α protein expression, *n* = 5; (G–J) Relative protein levels of USP9X, NOBOX, and HIF1α, *n* = 3. Mean ± S.E.M. ^*^ *p* < 0.05, ^**^ *p* < 0.01 vs. control. USP9X, ubiquitin‐specific protease 9, X‐linked; HIF1α, hypoxia inducible factor‐1α; *Nobox*, newborn ovary homeobox gene; KEGG, kyoto encyclopedia of genes and genomes; PW, postnatal week; IF, immunofluorescence; GD, gestational day.

Next, we investigated the regulatory mechanism by which USP9X influences follicular development. Transcriptomic analysis of adult female offspring ovaries (PW12) revealed significant downregulation of the HIF1 signaling pathway and associated genes in the PAE group (Figure 5A,B). Ovarian single‐cell database (GSE232309) demonstrated co‐expression of *USP9X* and *HIF1α* in 32.35% of ovarian cells (Figure S5). Concurrently, PAE significantly reduced HIF1α protein levels in GD18 and PW12 ovaries (Figure 5C–F).

To further elucidate the biochemical interactions within this pathway, we utilized NIH3T3 cells (a murine embryonic fibroblast line that has cellular plasticity and high expression of *Nobox*) as a biochemical model system. Cells were treated with varying aspirin concentrations to assess its effects on the USP9X‐HIF1α protein complex (Figure S6). The 10 µm aspirin group exhibited marked reductions in USP9X, NOBOX, and HIF1α protein levels (Figure 5G–K), confirming aspirin's suppression of the USP9X/HIF1α/NOBOX axis in this biochemical model. Together with our in vivo and in vitro ovarian data, these biochemical assays in NIH3T3 cells support a model where PAE‐associated USP9X downregulation is correlated with enhanced HIF1α ubiquitination and decreased HIF1α/NOBOX protein levels, potentially contributing to the disruption of folliculogenesis observed in ovarian tissue.

**FIGURE 6:**
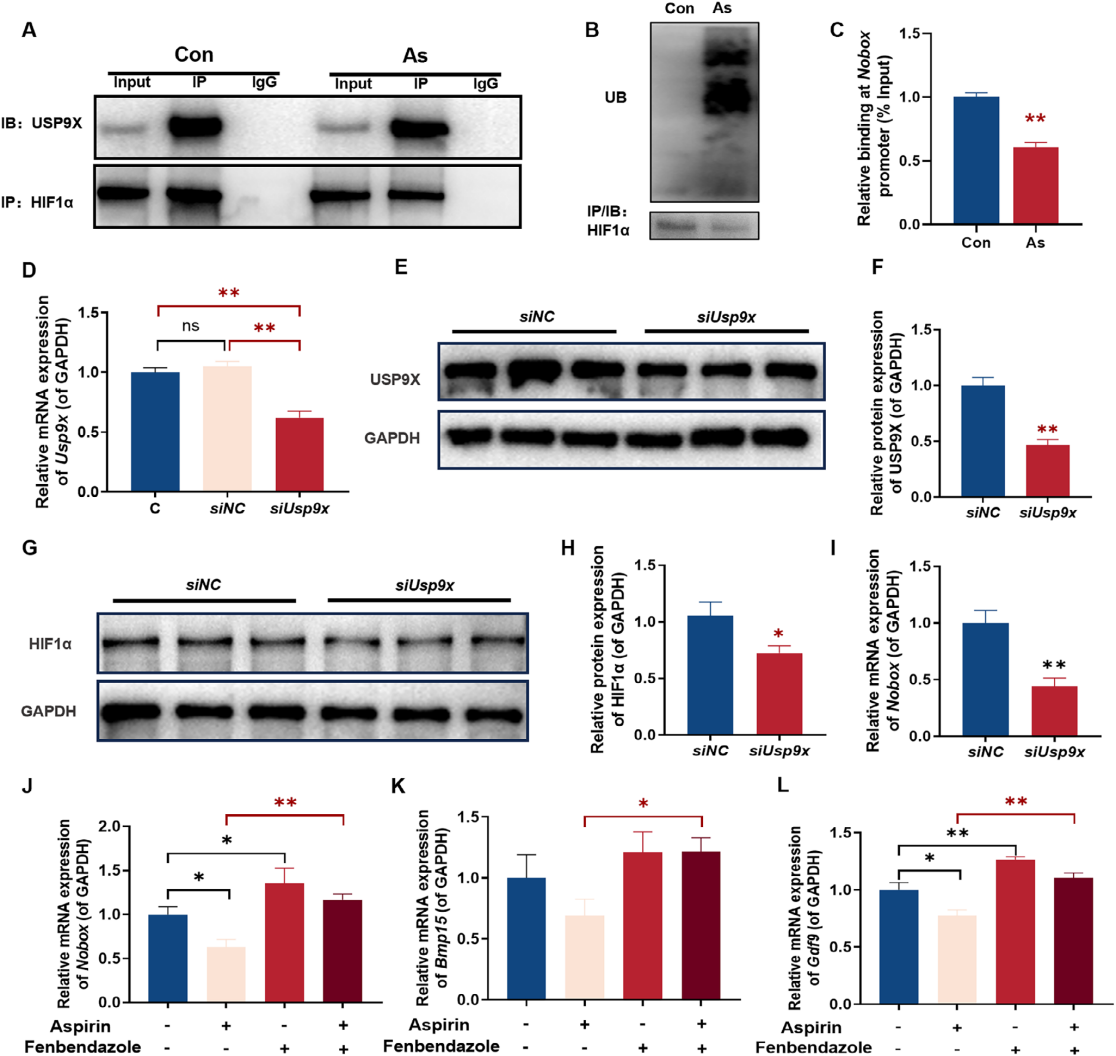
Changes of USP9X‐Hif1α‐NOBOX pathway in NIH3T3 cell line. (A) Protein–protein interactions between USP9X and HIF1α, *n* = 3; (B) Protein‐protein interactions between HIF1α and ubiquitin, *n* = 3; (C) Binding level of HIF1α on the *Nobox* promoter region, *n* = 5; (D) The relative mRNA expression of *Usp9x*, *n* = 6; (E) Protein expression of USP9X, *n* = 3; (F) The relative protein expression of USP9X, *n* = 3; (G, H) Relative protein levels of HIF1α, *n* = 3; (I) Relative mRNA expression of *Nobox*, *n* = 6; (J–L) Relative mRNA expression of follicular development‐related genes following treatment with fenbendazole, *n* = 6. Mean ± S.E.M. ^*^ *p* < 0.05, ^**^ *p* < 0.01 vs. control. USP9X, ubiquitin specific protease 9, X‐linked; HIF1α, hypoxia inducible factor‐1α; *Nobox*, newborn ovary homeobox gene; *Gdf9*, growth differentiation factor 9; *Bmp15*, bone morphogenetic protein 15.

To further delineate the interplay between USP9X, HIF1α, and NOBOX, we conducted mechanistic studies in NIH3T3 cells. Co‐IP experiments revealed that USP9X interacted with HIF1α, and compared to the control group, the binding between USP9X and HIF1α was reduced in the aspirin‐treated group (Figure 6A), accompanied by elevated HIF1α ubiquitination (Figure 6B). We designed five sets of tiling primers spanning the *Nobox* promoter region (Figure S7). ChIP‐PCR revealed diminished HIF1α binding to the *Nobox* promoter region in the aspirin group (Figure 6C). Following efficient USP9X knockdown in NIH3T3 cells via siRNA, which led to significant reductions of USP9X in both mRNA and protein levels (Figure 6D–F), we observed a consequent decrease in HIF1α protein level and Nobox mRNA expression (Figure 6G–I). To confirm HIF1α’s role in mediating aspirin‐induced suppression of NOBOX, we administered fenbendazole (1 µm), a HIF1α agonist. This treatment rescued aspirin‐induced downregulation of *Nobox*, *Bmp15*, and *Gdf9* (Figure 6J–L). Collectively, these in vitro biochemical data are consistent with the hypothesis that aspirin promotes HIF1α ubiquitination via USP9X suppression, which could lead to the inhibition of NOBOX and its downstream targets as seen in our fetal ovary models.

### 2.5 HDAC1 Activation is Linked to Aspirin‐Induced Epigenetic Dysregulation of *Usp9x*

**FIGURE 7:**
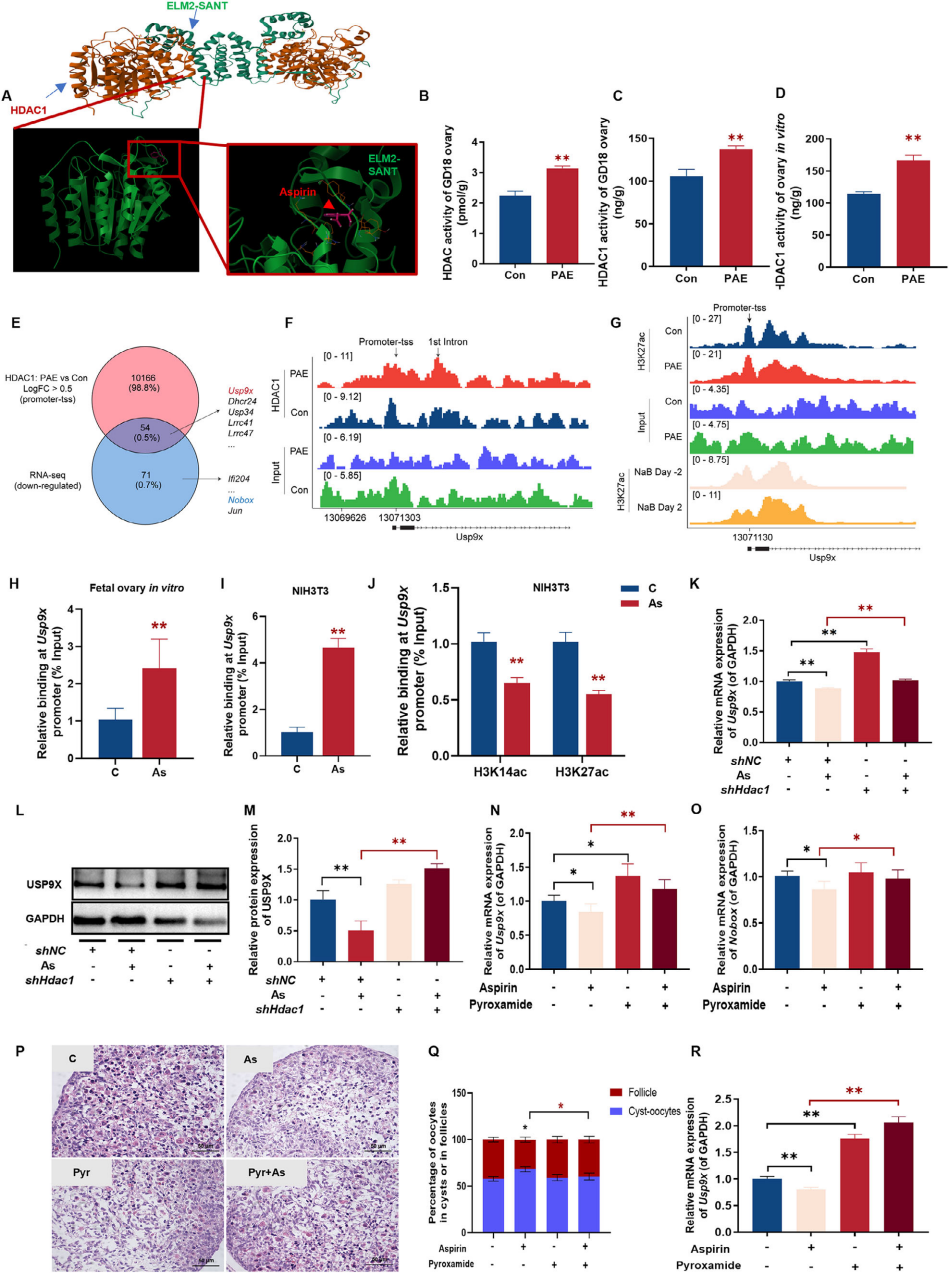
The change of HDAC1‐USP9X in offspring mice ovaries, in vitro cultured fetal ovaries, and NIH3T3 cell line. (A) Schematic diagram of molecular simulation docking between aspirin and HDAC1 protein complex using AutodockVina 1.2.2; (B) Total HDAC enzymatic activity in fetal ovaries from PAE and control mice at GD18, *n* = 6; (C, D) HDAC1‐specific enzymatic activity in GD18 fetal ovaries (C) and fetal ovaries cultured in vitro (D), *n* = 6; (E) Overlap of RNA‐seq identified downregulated genes and HDAC1 ChIP‐seq peaks in PAE fetal ovaries; (F) HDAC1 ChIP‐seq tracks at the *Usp9x* promoter in PAE and control ovaries. Input signals are shown as background control; (G) H3K27ac ChIP‐seq profiles at the *Usp9x* promoter in PAE and control ovaries, compared with public NaB‐treated embryonic stem cell data (GSE193085); (H, I) The relative binding level of HDAC1 on *Usp9x* gene promoter regions in NIH3T3 (H, *n* = 6) and fetal ovaries cultured in vitro (I, *n* = 3); (J) H3K14ac and H3K27ac levels at the *Usp9x* promoter in NIH3T3 cells by ChIP‐qPCR, *n* = 6; (K) The relative mRNA expression of *Usp9x*, *n* = 6; (L, M) The relative mRNA and protein level of USP9X, *n* = 3; (N) The relative mRNA expression of *Usp9x* in NIH3T3 cells, *n* = 6; (O) The relative mRNA expression of *Nobox*, *n* = 6; (P) Ovarian morphology by H&E staining, *n* = 6; (Q) The percentage of oocytes in cysts of in follicles, *n* = 5; (R) The relative mRNA expression of *Usp9x* in fetal ovaries cultured in vitro, *n* = 6. Mean ± S.E.M., *n* = 6. ^*^p< 0.05, ^**^p< 0.01 vs. control. Usp9x, ubiquitin‐specific protease 9, X‐linked; HDAC1, histone deacetylase 1; PAE, prenatal aspirin exposure; H3K14ac, histone 3 Lysine 14 acetylation; Nobox, newborn ovary homeobox gene.

Finally, we investigated the molecular mechanism underlying aspirin‐induced *Usp9x* suppression. Bioinformatic screening of the *Usp9x* promoter using the MOTIF database identified histone deacetylase 1 (HDAC1) as a candidate transcriptional regulator (Figure S8A). Molecular docking simulations (AutoDock Vina) further suggested a stable interaction between aspirin and the HDAC1 protein, with a favorable binding energy of −9.23 kcal/mol (Figure 7A). To functionally validate HDAC1 activation under aspirin exposure, we assessed HDAC enzymatic activity across our experimental models. Strikingly, PAE induced a significant increase in total HDAC enzymatic activity in PAE GD18 fetal ovaries (Figure 7B). The HDAC1 enzymatic activity was significantly elevated in both PAE GD18 fetal ovaries and fetal ovaries cultured in vitro after aspirin exposure (Figure 7C,D). This provided functional evidence that aspirin exposure is associated with HDAC1 activation.

To determine whether this HDAC activation is associated with direct epigenetic repression, we performed chromatin immunoprecipitation sequencing (ChIP‐seq) for HDAC1 and the active enhancer mark H3K27ac in fetal ovaries. Quality control analysis confirmed the high specificity of our ChIP‐seq libraries, with heatmaps demonstrating strong signal enrichment at the transcription start sites (TSS) of annotated genes relative to input controls (Figure S9A) and peak distribution analysis showing significant occupancy at promoter regions (Figure S9B). We hypothesized that direct targets of aspirin‐induced silencing would exhibit both transcriptional downregulation and enhanced HDAC1 binding. To identify these targets, we intersected the set of genes down‐regulated in PAE ovaries (identified via RNA‐seq) with genomic regions showing significantly increased HDAC1 binding (ChIP‐seq logFC > 0.5). This integrated analysis identified *Usp9x* as the top candidate gene exhibiting this overlapping signature (Figure 7E), suggesting it may be a preferential target of HDAC1‐mediated repression in this context. Notably, although genes such as *Nobox*, *Sohlh1*, *Gdf9*, and *Bmp15* were transcriptionally downregulated, their promoters showed no differential HDAC1 binding (Table S2), indicating that *Usp9x* is a specific direct target of HDAC1‐mediated repression, while other genes are likely affected through downstream or indirect mechanisms. Visualization of the genomic landscape at the *Usp9x* locus confirmed a specific enrichment of HDAC1 at the promoter region in PAE ovaries compared to controls (Figure 7F). Consistent with HDAC1‐mediated deacetylation, this increase in HDAC1 occupancy was accompanied by a concurrent reduction of the active histone mark H3K27ac at the same promoter region (Figure 7G). To further contextualize this finding, we compared our H3K27ac profiles with public datasets (GSE193085) derived from embryonic stem cells treated with Sodium Butyrate (NaB), a potent HDAC inhibitor. As expected, NaB treatment, which inhibits HDAC activity, resulted in increased H3K27ac signal. In contrast, our PAE samples mirrored the low‐acetylation state of untreated cells or inputs, reinforcing the inverse relationship between HDAC1 recruitment and H3K27ac deposition at the *Usp9x* promoter.

To validate these genome‐wide findings, we designed five sets of tiling primers spanning the *Usp9x* promoter region (Figure S10). ChIP‐qPCR analysis confirmed a significant enrichment of HDAC1 across these promoter regions in both fetal ovaries under in vitro culture and NIH3T3 cells. Concordantly, a significant reduction in H3K14ac and H3K27ac enrichment was observed in NIH3T3 cells following aspirin treatment (Figure 7H–J). Collectively, these data demonstrate that PAE is associated with the specific recruitment of HDAC1 to the *Usp9x* promoter, resulting in the erasure of H3K27ac marks and subsequent transcriptional silencing.

Furthermore, to determine the role of HDAC1 in aspirin‐induced suppression of USP9X expression, we knocked down HDAC1 expression using shRNA in NIH3T3 cells (Figure S11). The results showed that *Hdac1* knockdown reversed the inhibition of *Usp9x* mRNA and protein expression caused by aspirin in NIH3T3 cells (Figure 7K–M). Similarly, using the HDAC1‐specific inhibitor pyroxamide reversed the aspirin‐induced inhibition of *Usp9x* and *Nobox* mRNA expression (Figure 7N,O). In vitro fetal ovarian cultures further validated these findings: aspirin‐treated ovaries exhibited disrupted morphology and reduced follicular oocyte ratios, while pyroxamide co‐treatment restored normal ovarian architecture and elevated *Usp9x* expression (Figure 7P–R). Collectively, these results indicate that aspirin is associated with HDAC1 activation to suppress H3K27ac levels at the *Usp9x* promoter, driving its transcriptional repression.

## 3 Discussion

As an NSAID, aspirin has been increasingly utilized during pregnancy for both analgesia and prevention of obstetric complications. However, its potential impacts on offspring ovarian development and long‐term reproductive function have not been previously investigated. In this study, we established PAE mouse models based on clinically recommended dosing regimens. Current international guidelines suggest administering 100–162 mg/d aspirin from gestational weeks 12–16 through week 36 for preeclampsia prevention in high‐risk pregnancies [23], while the Chinese Society of Obstetrics and Gynecology recommends a preventive dose of 50–150 mg/d. Through interspecies dose conversion calculations [24], we determined that the human equivalent dose of 100 mg aspirin (for a 60 kg individual) corresponds to approximately 20.56 mg/kg·d in mice. Consequently, pregnant mice received oral gavage of 5, 10, or 20 mg/kg·d aspirin from gestational days 9 to 18. Notably, only the 20 mg/kg·d PAE group exhibited significant reductions in ovarian reserve and adult fertility, manifesting as decreased primordial and antral follicle counts during adolescence (postnatal week 6), accelerated follicular atresia in adulthood (postnatal week 12), and impaired superovulation outcomes with reduced embryo implantation and live birth rates. The observation that this significant morphological disruption was evident specifically at 20 mg/kg·d suggests the existence of a biological threshold. We propose that the ovarian developmental program possesses a certain buffering capacity, whereby the transcriptional network can compensate for minor perturbations induced by lower doses (5–10 mg/kg·d). However, beyond a critical level of disruption, these compensatory mechanisms fail, leading to the overt failure of primordial follicle assembly. This threshold effect is consistent with the robustness observed in many developmental systems and reframes the dose‐response relationship from a purely linear model to one acknowledging nonlinear, critical‐state dynamics. Importantly, the 5–10 mg/kg·d doses showed no adverse effects on oocyte development or primordial follicle assembly, delineating a critical safety threshold for clinical reference. In summary, our study descriptively characterizes the dose‐related impact of PAE, identifying a high‐dose threshold for overt ovarian pathology and associated molecular changes, without evidencing a proportional epigenetic disruption across the entire lower dose range.

DOR, a condition characterized by quantitative and qualitative declines in the primordial follicle pool, is increasingly recognized as a fetal‐origin disorder influenced by intrauterine environmental insults [25]. Our findings demonstrate that PAE disrupts primordial follicle assembly during late gestation (GD18) and early postnatal stages (PD3), marked by reduced incorporation of oocytes into primordial follicles and persistent downregulation of key folliculogenesis regulators, including NOBOX, SOHLH1, GDF9, BMP15, and the KIT/KITL system. These factors, which are predominantly expressed in oocytes and granulosa cells across follicular developmental stages, orchestrate primordial follicle formation through complex intercellular crosstalk [26, 27, 28]. The observed histopathological alterations, such as disorganized germ cell nests, nuclear pyknosis in cortical oocytes, and reduced follicular oocyte ratios, further substantiate the fetal developmental origins of PAE‐induced ovarian dysfunction.

Emerging evidence highlights the pivotal role of epigenetic reprogramming in mediating fetal‐origin reproductive disorders [29]. Through transcriptomic profiling of PAE offspring ovaries, we identified profound dysregulation in protein deubiquitination pathways, with USP9X, a deubiquitinating enzyme essential for germ cell survival and follicular development [30, 31, 32], emerging as a candidate mediator. The sustained suppression of USP9X expression from GD18 through PW12 correlated strongly with impaired NOBOX signaling, a transcriptional master regulator of folliculogenesis. Although the specific function of USP9X in primordial follicle assembly remains largely unknown, prior evidence strongly suggests its critical, non‐redundant role in the female reproductive system. USP9X exhibits stage‐ and sex‐dependent expression during oogenesis, being highly expressed in primordial and primary follicle granulosa cells and oocytes in mature follicles [33]. Furthermore, recent mechanistic studies have confirmed its active role in follicular health, demonstrating that USP9X prevents granulosa cell apoptosis and ensures follicular survival by directly stabilizing the TGFBR2 receptor, thereby activating the TGF‐β signaling pathway [34]. Moreover, clinical data links USP9X to female development, as loss‐of‐function mutations cause syndromic disorders with congenital malformations [35]. Based on this foundation and our own robust sequencing data, we focused on USP9X as a candidate upstream regulator. Mechanistic studies revealed that USP9X stabilizes HIF1α, a hypoxia‐inducible factor critical for follicular maturation [36, 37, 38]. PAE‐induced USP9X deficiency promoted HIF1α ubiquitination and proteasomal degradation, thereby attenuating its binding to the NOBOX promoter and subsequent activation of downstream folliculogenic genes. Rescue experiments using HIF1α agonists confirmed the reversibility of this pathway, supporting the USP9X‐HIF1α‐NOBOX axis as a potential regulatory mechanism. These data indicate a potential pathway, but further mechanistic work is required.

The epigenetic landscape, particularly histone acetylation dynamics, plays a decisive role in ovarian developmental programming [39, 40]. Moving beyond molecular docking predictions, our functional assays confirmed that aspirin significantly increases total HDAC and HDAC1‐specific enzymatic activity in vivo within fetal ovaries. Crucially, our comprehensive ChIP‐seq profiling resolved the question of target specificity: while HDAC activity is broadly upregulated, HDAC1 is specifically recruited to the *Usp9x* promoter, but not to the promoters of downstream genes like *Nobox* or *Sohlh1*. This specific recruitment leads to reduced H3K14ac and H3K27ac levels at the *Usp9x* promoter. This epigenetic silencing of USP9X transcription was functionally validated through HDAC1 knockdown and pharmacological inhibition experiments, which successfully restored USP9X expression and primordial follicle assembly in the PAE‐induced DOR model. Our findings suggest that aspirin exposure is associated with epigenetic changes at the *Usp9x* promoter and alterations in downstream oocyte regulators, proposing HDAC1 inhibition as a potential therapeutic strategy to mitigate PAE‐associated ovarian dysfunction.

This study has certain limitations. First, the use of NIH3T3 fibroblasts to interrogate the USP9X‐HIF1α‐NOBOX axis, while enabling controlled biochemical and epigenetic assays, cannot recapitulate the oocyte‐specific chromatin context. Therefore, findings from this cell line should be interpreted as supporting biochemical interactions rather than modeling germ cell biology. To address this, we performed parallel validation in an in vitro fetal ovary culture system, providing complementary evidence for PAE‐induced alterations in this pathway. Second, while this study identifies a candidate molecular pathway in a preclinical model, species differences in physiology and the focus on a relatively high dose necessitate caution in extrapolation to humans. Therefore, further clinical and epidemiological studies are necessary to evaluate its potential relevance to human pregnancy outcomes and ovarian health.

Collectively, our findings suggest that prenatal aspirin exposure is associated with HDAC1‐linked epigenetic alterations at the *Usp9x* promoter and downstream impairment of the HIF1α/NOBOX axis, identifying a candidate pathway for fetal‐origin DOR. While these data indicate a potential mechanistic framework, further work is required to fully establish causality and component interactions in vivo. Ultimately, these insights advance our mechanistic understanding of how prenatal drug exposure impacts fetal ovarian development and provide a preclinical foundation to guide future research on ovarian reserve preservation.

## 4 Conclusion

**FIGURE 8:**
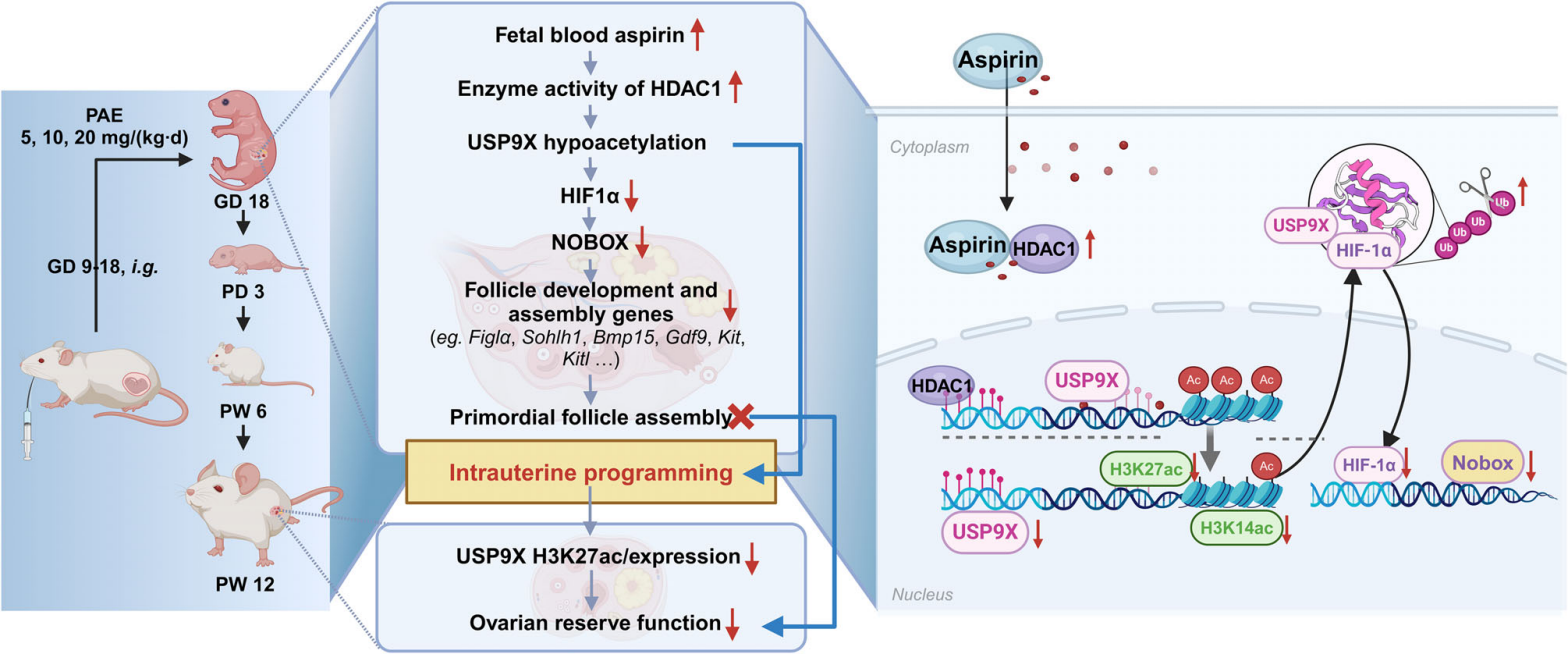
Mechanism diagram.

This study provides experimental evidence that PAE at 20 mg/kg·d (equivalent to clinical dosing regimens) induces ovarian developmental abnormalities, DOR, and fertility impairment in female offspring. The underlying mechanism involves a functional elevation of HDAC1 enzymatic activity in fetal ovaries. Epigenomic profiling further reveals that this activated HDAC1 is specifically recruited to the *Usp9x* promoter, which reduces histone acetylation levels (H3K14ac and H3K27ac) and suppresses its expression. This specific epigenetic dysregulation enhances HIF1α ubiquitination and degradation, ultimately leading to downregulation of NOBOX and its downstream folliculogenesis‐related genes, coupled with inhibition of primordial follicle assembly. Critically, these epigenetic alterations associated with USP9X persist postnatally, perpetuating follicular developmental suppression and culminating in adult ovarian reserve depletion and reproductive dysfunction (Figure 8). Our findings implicate USP9X‐associated dysregulation as a plausible and significant contributor to PAE‐induced ovarian pathologies. This study provides experimental evidence that PAE at a clinically relevant high dose can impair ovarian development in mice through a mechanism associated with HDAC1‐USP9X‐HIF1α‐NOBOX axis dysregulation. The persistence of these molecular alterations postnatally suggests a potential for long‐term reproductive consequences. Collectively, this work offers a valuable preclinical model and identifies a candidate mechanistic pathway, thereby adding to the essential evidence base required for the ongoing, comprehensive assessment of medication safety in pregnancy.

## 5 Experimental Section

### 5.1 Chemicals

Aspirin (CAS No: 117772‐70‐0, Item No: MB1024‐2) was obtained from Dalian Meilun Biotechnology Co., LTD. (Dalian, Liaoning, China). Isoflurane (No. R510‐22) was purchased from RWD Life Science Co., Ltd. (Shenzhen, Guangdong, China). Mice estradiol (E2, No. AR E‐8800) enzyme‐linked immunosorbent assay (ELISA) kit was obtained from Beijing North Institute of Biological Technology Co., Ltd. (Beijing, China). TRIzol^TM^ Reagent (No. 15596026) was procured from Thermo Fisher Scientific Co., Ltd. (Waltham, MA, U.S.A). Cham Q^TM^ Universal SYBR qPCR Master Mix (No. Q712‐02) and HiScript III RT SuperMix for qPCR (+gDNA wiper) (No. R323‐01) were procured from Vazyme Biotech Co., Ltd. (Nanjing, Jiangsu, China). The primers for reverse transcription real‐time quantitative polymerase‐chain‐reaction (RT‐qPCR) were customized and synthesized by Wuhan Tianyi Huayu Gene Technology Co., Ltd. (Wuhan, Hubei, China). The antibodies of MVH (No. A15624), BMP15 (No. A7321), USP9X (No. A9782), and HDAC1 (No. A19571) were purchased from ABclonal Technology Co., Ltd. (Wuhan, China). The antibodies of NOBOX (No. sc‐514178), HIF1α (No. sc‐13515), and Ubiquitin (No. sc‐8017) were purchased from SANTA CRUZ BIOTECHNOLOGY, INC. (Wuhan, China). All the primers were synthesized by TIANYIHUIYUAN Biotechnology Co., Ltd. (Wuhan, China). Other chemicals and reagents were of analytical grade.

### 5.2 Animals and Treatment

Animal experiments were conducted at the Center for Animal Experiments, Wuhan University, accredited by AAALAC International. All procedures were approved by the Experimental Animal Welfare Ethics Committee of Zhongnan Hospital, Wuhan University (permit number: ZN2022228), following NIH regulations and ARRIVE guidelines. Eight‐week‐old Kunming mice were obtained from Beijing Speifu Biotechnology Co., Ltd. and housed at 20°C–24°C with 50%–65% humidity. Mice were acclimated and mated, with gestational day (GD) 0 marked by sperm or a vaginal plug. Pregnant mice were randomly assigned to control or PAE groups. From GD9 to GD18, the PAE group received 20 mg/kg.d aspirin, while controls received saline.

In‐utero experiment: Pregnant mice were given either saline or aspirin from GD9 to GD18. On GD18, 1 h after the final gavage, mice were anesthetized and euthanized. A cesarean section was performed to count live pups, with litters containing 8–14 pups. Pups were sexed, euthanized, and ovarian tissues from female pups were collected. Five pregnant mice from each group had one ovary from a selected pup taken for analysis. Remaining ovaries were frozen, and serum was stored at −80°C.

Postnatal experiment: Pups were born naturally, and their number was recorded. Mothers with 12–18 pups were retained, and the number of female pups was adjusted to six per litter. At 4 weeks (PW4), pups were weighed and weaned. Mothers were euthanized, and offspring were grouped into PW6, PW12, and PW12 chronic stress groups (*n* = 10 for each group). For the chronic stress groups, female offspring mice from different litters were used (*n* = 10). Mice in the stress groups underwent a 2‐week chronic stress protocol from PW10 to PW12. The model involved forced ice‐water swimming (4°C, 5 min/day, for 14 consecutive days), which is an established method for inducing chronic stress. After the euthanasia of offspring, the left ovaries of five mice of each group were fixed with paraformaldehyde for morphological analysis, and the rest were frozen for further experiments. Serum was collected and stored at −80°C. Additionally, some PW12 offspring were caged with normal male mice, and pup birth rates, live birth, and stillbirth rates were observed.

### 5.3 Hematoxylin‐Eosin (H&E) Staining

Fetal ovaries were fixed in 4% paraformaldehyde overnight, routinely stained with hematoxylin–eosin (H&E), the maximum cross‐section of the fixed ovaries in each group was sectioned by the Paraffin sectioning technique, then observed and photographed with an Olympus AH‐2 light microscope (Olympus, Tokyo, Japan). Another experimenter conducted the examination and evaluation blindly. We photomicrograph and evaluated relevant examination indexes (such as the maximum cross‐sectional area and diameter of fetal ovaries) from each section (*n* = 5). To classify and count oocytes, we selected 5 unit squares of interstitial tissue areas per section randomly and calculated the number of oocytes per unit square by Image J analysis. The oocytes were counted in five different sections; every section was 4 × 10^4^ µm^2^. For follicle quantification, ovarian tissues were serially sectioned at 5 µm, with four consecutive sections mounted per slide. Every fifth slide was selected for H&E staining. From each stained slide, one ovarian section was used to count follicles containing clearly visible oocytes. The counts from these systematically sampled sections were summed to estimate the total follicle number per ovary. Follicles were classified as follows: primordial follicles consisted of an oocyte surrounded by a single layer of flattened pregranulosa cells; primary follicles had a single layer of cuboidal granulosa cells; secondary follicles contained multiple layers of granulosa cells without an antrum; antral follicles exhibited antral formation. Atretic follicles were identified by disrupted follicular morphology, along with nuclear pyknosis, lysis, or fragmentation of granulosa cells.

### 5.4 Ovulation Induction

F1 female mice were raised to 12 weeks of age, and their estrous cycles were monitored using vaginal smears. Mice in estrus (*n* = 6) were selected for ovulation induction. The induction protocol involved administering 0.5 mL PMSG (200 IU/mL) via intraperitoneal injection, followed by a 0.3 mL hCG (400 IU/mL) injection 48 h later. Twelve to fourteen h after the hCG injection, the mice were anesthetized and euthanized. The ovaries and oviducts were exposed, and the oviducts were placed in M2 culture medium. Under a dissecting microscope, the ampullary region of the oviduct was punctured to release cumulus‐oocyte complexes (COCs), which were then transferred to a hyaluronidase solution for 5 min. The oocytes floated to the surface, and the granulosa cells sank. The oocytes were carefully collected, washed 2–3 times in fresh M2 medium, and counted and photographed under a microscope.

### 5.5 Hormonal Level Measurements

Serum levels of estradiol (E2), progesterone (P), follicle‐stimulating hormone (FSH), luteinizing hormone (LH), and anti‐Müllerian hormone (AMH) were measured using ELISA kits, following the manufacturer's protocol. Serum from female mice was thawed on ice and mixed. The ELISA kits were equilibrated at room temperature for 20 min. Standard and sample wells were prepared, with standard solutions (S0‐S5) added to the standard wells and 10 µL of serum and 40 µL of sample diluent added to the sample wells. Then, 100 µL of HRP‐labeled detection antibody was added to each well. The plate was sealed and incubated at 37°C for 60 min. After discarding the liquid, wells were washed five times with wash buffer. Substrates A and B (50 µL each) were added and incubated at 37°C in the dark for 15 min. After adding 50 µL of stop solution, the optical density (OD) was measured at 450 nm using a microplate reader. A standard curve was created by plotting standard concentrations against OD values, and the concentrations of each sample were determined based on the curve.

### 5.6 Total RNA Extraction, Reverse Transcription, and RT‐qPCR for Ovary

Total RNA was extracted from the ovaries using the TRIzol ^TM^ Reagent following the manufacturer's protocol. The tissues of each littermate were pooled for homogenization as one sample. The concentration and purity of the total RNA were determined using a spectrophotometer (NanoDrop 2000), and the total RNA concentration was adjusted to 1 mg/mL. Single‐strand cDNA was prepared from 1 mg of total RNA according to the protocol of the kit and was stored at −20°C until use. All the primers were designed using Primer Premier 5.0 (PREMIER Biosoft International, CA). The sequences of each of the designed primers were queried using the NCBI BLAST database for homology comparison and are listed in Table 1. The RT‐qPCR was conducted with QuantStudio 5 Real‐Time PCR System (Thermo Fisher Scientific, Waltham, MA, USA) using ChamQ Universal SYBR qPCR Master Mix. To quantify the gene transcripts more precisely, the mRNA level of the housekeeping‐gene glyceraldehyde 3‐phosphate dehydrogenase (GAPDH) was measured and used as a quantitative control. Each sample was normalized according to GAPDH mRNA levels by the 2^−△△Ct^ method.

**TABLE 1:** Oligonucleotide primers and PCR conditions in real‐time quantitative PCR.

Genes	Forward primer (5’‐3’)	Reverse primer (5’‐3’)	Annealing (°C)
*Gapdh*	GCAAGTTCAATGGCACAG	GCCAGTAGACTCCACGACA	57.8
*Nobox*	GACATGGGACCTCAGGATTA	GAGTCTTCTGGTGGTAGAAATG	57.6
*Figlα*	AGAGCGTGAGCGGATAAA	CCAGAACACAGCCAAGTATC	57.4
*Sohlh1*	TCTGATGACCACCAATCCTGACTG	CATCCACTGGGCTGCTCCTG	55.9
*Gdf9*	GCCCGTCCCGTGAAAGAGG	GCTGAGGTTGAAGGATGCTGTAAG	57.1
*Bmp15*	GTGCTCAGGCTAAACTTCTT	GGAGGGAACACTGGTTATTT	59.5
*Kit*	TCTGCTCTGCGTCCTGTTGG	GATTGTGCTGGATGGATGGATGG	57.8
*Kitl*	TCTGCGGGAATCCTGTGACTG	ACCATATCTCGTAGCCAACAATGAC	57.6
*Col20a1*	ACGCCACCTCATCCAGTATCC	CCTCCACCACCACCTCCTTG	55.6
*Col27a1*	GCTGGCATCAGACACTGTTCAC	TCAGAGGAAGCACGCAGGTC	55.9
*Col11a1*	CCTATGAGATGCCTGCGTTTACTG	GTCTGCGGATTGTAGTTCTGTCTG	57.1
*Col9a1*	GCCAGGAAGACAAGGACACAAG	CAACTATGCCAGTGATGCCTCTC	59.4
*Sf1*	CCAGTACGGCAAGGAAGA	GAGGCTGAAGAGGATGAGGA	55.9
*Star*	GGGAGATGCCTGAGCAAAGC	GCTGGCGAACTCTATCTGGGT	59.4
*Cyp11a1*	GCTGCCTGGGATGTGATTTTC	GATGTTGGCCTGGATGTTCTTG	57.6
*Cyp17a1*	ACTATCCGAGAAGTGCTGCGTAT	GCTCCGAAGGGCAAGTAACTC	55.3
*3β‐hsd*	GGGTATTCTGTGTGTTACTGG	ACTGTCCTTGGATGCTTGTAG	55.6
*Cyp19a1*	ATGGGCCTCCTTCTCCTGAT	CAGGCACTTCCAATCCCCAT	55.6
*Usp9x*	GTTCTGAGGATGAAGAGTGGCTTAC	CTGTGGTTGATGAAGGCTATCTCG	55.9

*Gapdh*, glyceraldehyde 3‐phosphate dehydrogenase; *Nobox*, newborn ovary homeobox gene; *Figlα*, factor in the germline alpha; *Sohlh1*, spermatogenesis and oogenesis specific basic helix‐loop‐helix 1; *Gdf9*, growth differentiation factor 9; *Bmp15*, bone morphogenetic protein 15; *Kit*, receptor tyrosine kinase; *Kitl*, kit ligand; *Col20a1*, collagen type XX alpha 1 chain; *Col27a1*, collagen type XXVII alpha 1 chain; *Col11a1*, collagen type XI alpha 1 chain; *Col9a1*, collagen type IX alpha 1 chain; *Sf1*, steroidogenic factor 1; Star, steroidogenic acute regulatory protein; *Cyp11a1*, cytochrome P450 family 11 subfamily A member 1; *Cyp17a1*, cytochrome P450 family 17; *3β‐hsd*, hydroxysteroid 3‐beta dehydrogenase 1; *Cyp19a1*, cytochrome P450 family 19 subfamily A member 1; *Usp9x*, ubiquitin specific peptidase 9, X‐linked.

### 5.7 Immunofluorescence Staining (IF)

The paraffin‐embedded ovary tissue was cut into 5 µm sections, deparaffinized in xylene, and rehydrated. The sample was repaired in a citrate buffer (10 mm, pH 6.0) for 5 h at a constant temperature of 65°, and rinsed with PBS 3 times. After blocking in 5% BSA for 30 min at room temperature, the sections were then incubated overnight at 4°C with the primary antibody: anti‐MVH (1:100), anti‐PCNA (1:100), followed by subsequent incubation with the fluorescent secondary antibody (1:200) for 2 h at room temperature, and stain the sections with 4',6‐diamidino‐2‐phenylindole (DAPI) (1 µg/mL) for 10 min. All images were captured using an Olympus AH‐2 Light Microscope (Olympus, Tokyo, Japan). Analysis of the stained images was performed using Olympus software. Quantification of fluorescence intensity was performed using ImageJ (Fiji). To ensure unbiased quantification and accurate delineation of oocytes, we utilized the Trainable Weka Segmentation plugin. A pixel‐based classification model was trained using a Random Forest algorithm to distinguish oocytes from ovarian stromal tissue and background based on distinct morphological features (rounded shape) and texture. This automated segmentation generated binary masks defining oocyte‐specific Regions of Interest (ROIs). These masks were applied to the raw, unmodified fluorescent channels for MVH, NOBOX, HIF1a, and USP9X. The Mean Fluorescence Intensity (MFI) was calculated exclusively within the masked oocyte cytoplasm, ensuring that non‐specific background signal was strictly excluded from the analysis. Data were compiled from 5 independent biological replicates.

### 5.8 Sequencing and Bioinformatics Analysis

Sample Collection and Sequencing:Ovarian tissues from three litters of fetal mice were collected for transcriptome sequencing by Beijing Novogene Technology Co., Ltd. The process included: a. RNA Extraction and Quality Check: RNA was extracted from fetal ovaries and assessed for integrity and quantity using an Agilent 2100 bioanalyzer. b. Library Construction and Quality Control: At least 1 µg of total RNA was used to construct the sequencing library. PolyA‐tailed mRNA was enriched using oligo(dT) beads, fragmented, and converted into cDNA. The cDNA was purified, repaired, and ligated with adapters. AMPure XP beads were used to select cDNA fragments (370–420 bp), followed by PCR amplification and quality checks. c. Sequencing: The qualified libraries were pooled and sequenced using Illumina technology to generate 150 bp paired‐end reads. Raw data were filtered to remove low‐quality reads and adapter sequences. HISAT2 was used to align the reads to the reference genome, and FeatureCounts calculated gene expression levels.

Bioinformatics analysis: Differential expression analysis was performed using DESeq2, applying statistical methods with thresholds set at *p* < 0.05 and |log_2_ fold change| > 1. Only samples with RNA integrity values ≥ 8.0 were sequenced on the Illumina HiSeq platform. Raw reads were aligned to the mouse reference genome (mm10) using the STAR aligner. Gene Ontology (GO) and KEGG pathway enrichment analyses were done using ClusterProfiler software, with results visualized using the enrichplot function to highlight relevant pathways associated with differentially expressed genes.

### 5.9 Ovarian Culture In Vitro

#### 5.9.1 Ovary Culture Medium Preparation

The α‐MEM medium and DMEM/F12 medium were mixed in a 1:1 ratio to create a base medium. Fetal bovine serum (final concentration 5%), penicillin‐streptomycin solution (final concentration 0.5%), and sodium pyruvate (final concentration 0.23 mmol/L) were sequentially added to prepare a complete culture medium. The pH was adjusted to 7.2–7.4 with 7.5% NaHCO_3_ and filter‐sterilized (0.22 µm).

#### 5.9.2 Fetal Ovary Collection and Culture

After performing a cesarean section on untreated GD18 pregnant mice, female pups were taken and immersed in 75% alcohol for 3–5 s, then quickly transferred to a sterile workbench and immersed in 75% alcohol again for 3–5 s. Under sterile conditions, sterilized fine tweezers were used to open the abdominal cavity of the female pups, and using another sterilized fine tweezer, the bilateral ovaries were quickly removed after locating the kidneys and uterus. The ovaries were washed three times with PBS and placed in a pre‐prepared 24‐well culture plate, with a maximum of three ovaries per well. One mL of the specially prepared ovarian culture medium was added to each well. The plate was then placed in a constant‐temperature incubator (37°C, 5% CO_2_) for culture.

#### 5.9.3 Experimental Treatments In Vitro

1. Aspirin Treatment: For dose‐response experiments, aspirin (1, 10, or 100 µm) was added to the culture medium at the beginning of the culture (Time 0 h) and incubated for 48 h.
2. HDAC1 Inhibitor Treatment: For rescue experiments, ovaries were treated with Aspirin (10 µm), Pyroxamide (1 µm), or a co‐treatment of both for 48 h.
3. Sample Collection: At the 48 h endpoint, ovarian tissues were harvested and fixed for H&E staining or snap‐frozen for RNA extraction (RT‐qPCR).

### 5.10 Cell Experiments

#### 5.10.1 Cell Culture and Viability Assay

NIH3T3 cells were obtained from Punoce and cultured in complete medium (DMEM with 10% fetal bovine serum and 1% antibiotics) under 5% CO_2_ at 37°C. Cells were passaged and handled when they reached approximately 90% confluence. NIH3T3 cells were treated with aspirin at concentrations of 1, 10, 100, and 1000 µm for various durations (24, 48, and 72 h). After treatment, cell viability was assessed using the MTS assay. The old medium was discarded, and a diluted MTS solution was added to each well. The plate was incubated in the dark at 37°C for 15–30 min, and the absorbance at 490 nm was measured for analysis.

#### 5.10.2 Cell Treatments

The cells were treated with aspirin at concentrations of 1, 10, and 100 µm, with a control group receiving solvent only. After aspirin treatment, cells were washed and cultured in low serum medium for 48 h. Additionally, NIH3T3 cells were transfected with Usp9x siRNA using Lipo3000, followed by culture in low serum medium for 48 h. For HIF1α activation, cells were treated with 1 µm Fenbendazole or a combination of Fenbendazole and 10 µm aspirin, with cells collected after 48 h and medium replaced the next day.

### 5.11 Gene Knockdown and Transfection Procedures

Two knockdown approaches were employed based on experimental requirements: transient *Usp9x* siRNA knockdown for acute functional assays and stable *Hdac1* shRNA knockdown to maintain gene suppression during prolonged drug treatment.

#### 5.11.1 *Usp9x* siRNA Transfection in Fetal Ovaries

Fetal ovaries were equilibrated in medium for 30 min at 37°C. For each well of a 24‐well plate, Lipofectamine 3000 (2 µL) and *Usp9x* siRNA (20 µm, 2 µL) were separately diluted in 100 µL serum‐free DMEM/F‐12, incubated for 5 min at RT, combined, and incubated for 20 min. The complex (200 µL) was added to the culture. After 6–8 h, the medium was replaced. Ovaries were cultured for 48 h with half‐medium changes every 24 h.

#### 5.11.2 *Usp9x* siRNA Transfection in NIH3T3 Cells

Cells were transfected with Usp9x siRNA or scrambled control (siNC) using Lipofectamine 3000. For a 6‐well plate, 10 µL siRNA (200 nm final) and 5 µL Lipofectamine 3000 were separately diluted in 125 µL serum‐free medium, incubated for 5 min, mixed, and incubated for 20 min. The mixture was added to cells in 1.75 mL serum‐free medium. After 6 h, the medium was replaced with low‐serum medium. Cells were harvested 48 h post‐transfection.

#### 5.11.3 *Hdac1* shRNA Transfection

Cells were transfected with shRNA plasmids (sequences in Table 2) using Lipofectamine 3000. For a 6‐well plate, 2.5 µg plasmid and 7.5 µL Lipofectamine 3000 were diluted in 125 µL serum‐free medium, incubated for 5 min, combined, and incubated for 20 min. The complex was added to the cells. After 6–8 h, the medium was replaced with low‐serum medium, supplemented with 10 µm aspirin for rescue experiments. Cells were harvested 48 h later.

**TABLE 2:** Sequences of siRNA and shRNA oligonucleotides used in this study.

Target Gene	Oligo Name	Sequence (5' to 3')	Type
*Usp9x*	siRNA Sense	ACACAACUCGACAGUCUCATT	siRNA
	siRNA Antisense	UGAGACUGUCGAGUUGUGUCC	siRNA
Control	siNC Sense	UUCUCCGAACGUGUCACGUTT	siRNA
	siNC Antisense	ACGUGACACGUUCGGAGAATT	siRNA
*Hdac1*	shRNA‐1	GAGGGCCTATTTCCCATGA	shRNA
	shRNA‐2	GCTTGGGTAATAGCAGCCATT	shRNA
	shRNA‐3	GCCAGTCATGTCCAAAGTAAT	shRNA

### 5.12 Western Blot (WB)

Cell and ovarian tissue protein extraction: Treated NIH3T3 cells are washed three times with PBS, then scraped from the culture dish and collected in a centrifuge tube. RIPA lysis buffer is added, and the mixture is incubated at 4°C for 30 min to 1 h for complete lysis. The lysate is centrifuged at 12 000 rpm for 15 min, and the supernatant is collected. For in vitro cultured fetal ovaries (eight per sample), 1 mL of IP lysis buffer (containing 1% PMSF) is added, and the mixture is ground and incubated at 4°C for 20 min. After centrifugation, the supernatant is transferred to RNase‐free tubes. In both cases, 5× loading buffer is mixed with the supernatant at a 4:1 ratio, heated at 100°C for 5 min, and stored at −80°C.

Protein quantification and western blotting: Protein standards are prepared according to the BCA kit instructions, dissolving to a concentration of 25 mg/mL. Various volumes of diluted protein standards are added to a 96‐well plate, along with BCA working solution, and incubated at 37°C for 30 min. OD values are measured, and sample protein concentrations are calculated using a standard curve. For Western blotting, glass plates are cleaned, and the gel is prepared. After electrophoresis, the membrane is transferred and blocked, followed by incubation with the target antibody and a secondary antibody. Detection is performed, and the relative optical density values of the bands are analyzed using image analysis software.

### 5.13 Co‐Immunoprecipitation (CoIP)

Cells are washed twice with PBS and lysed with RIPA buffer (200 µL per well for a 6‐well plate; 1.2 mL for a 75T flask) on ice or at 4°C for 30 min, mixing gently every 5 min. The lysate is scraped off, transferred to a 1.5 mL tube, and centrifuged at 14,000 rpm for 15 min at 4°C. The clear supernatant is reserved, and 100 µL is set aside for Input, with protein concentration measured by BCA. The remaining supernatant (400 µL each) is mixed with 1 µg of specific antibodies (USP9X, HIF1α, IgG), sealed, and incubated overnight at 4°C. Protein G/A beads are washed with PBS and resuspended in an equal volume to achieve a 50% concentration. 40 µL of the washed beads is added to each antibody‐incubated lysate and incubated at 4°C for 3–5 h. After centrifugation at 14 000 rpm for 20 s, the supernatant is discarded, and the beads are washed three times with 800 µL of lysis buffer. Finally, 20–30 µL of loading buffer is added, heated at 100°C for 5–10 min, and then centrifuged again before proceeding with Western blot analysis.

### 5.14 Chromatin Immunoprecipitation (ChIP)

NIH3T3 cells were cross‐linked with 1% formaldehyde for 13 min, and cross‐linking was stopped with 400 µL of glycine. Cells were then scraped, centrifuged, and washed. For chromatin shearing, cells were incubated with lysis buffer, sonicated, and centrifuged. The supernatant was collected, and 10 µL was saved as Input. Protein G/A beads were blocked, added to the lysate, and incubated overnight at 4°C. After washing, sonicated chromatin was incubated with HIF1α antibody or IgG (negative control) overnight at 4°C. Beads were washed and eluted with Elution Buffer containing proteinase K. The elution was collected twice, and DNA was de‐crosslinked and purified with proteinase K, followed by DNA extraction.

Primers for ChIP‐PCR assays were designed to target functionally relevant regions within the promoter, defined as the sequence spanning from 2000 bp upstream to 500 bp downstream of the transcriptional start site (TSS), which were retrieved from the UCSC Genome Browser. Potential binding sites or regulatory regions were prioritized using publicly available ChIP‐seq data from resources such as ENCODE to identify loci enriched for specific histone modifications (e.g., H3K27ac) or transcription factor binding signals. Genomic sequences of selected sub‐regions were retrieved from the UCSC Genome Browser, and primers were designed using Primer‐BLAST under the following criteria: amplicon sizes ranging from 75 to 150 bp, primer melting temperature of 55°C–60°C, and GC content of 40%–60%. All primer pairs were verified for specificity in silico using NCBI Primer‐BLAST and validated for specificity by ChIP‐PCR.Real‐time PCR was performed using primers for the mouse *Nobox* promoter region (Table 3) and the *Usp9x* promoter region (Table 4).

**TABLE 3:** *Nobox* promoter region amplification sequence (mouse).

Number	Forward	Reverse
1	CCTTGGCTTCCTAACTGTGC	TGCCCCAAACCTCCCTTAAT
2	TGTCCCGGTAGCTGATTGTT	TGGCCAGATTGAAACAGCTG
3	AAATGACCCCAGAGCCTTGA	GGGGTTTACTAGGCCTCACA
4	AATCAAAACACGTGAGCCCG	GGAATGTGAGTGTTGGCCAG
5	TTCAAGGGTCAAGAGGGGAG	CAGAACAGCAATGGAAGGGG

**TABLE 4:** *Usp9x* promoter region amplification sequence (mouse).

Number	Forward	Reverse
1	GGCTTGTCCTATCCTCGTGT	GACTAGATTCCTGGGTCCCG
2	TCAAAGTGGAGTGGGAAGCA	ACACGAGGATAGGACAAGCC
3	TCAGTCCCAGTCACTATGCC	TGCTTCCCACTCCACTTTGA
4	TAGCATTGTCTTCCCCTCCC	GGGAAAGAAAGGAAGAGCGC
5	AAATGACTGTGTTGGGCTGG	GGGGATTGAACTCAGGAGCT

The Input is used as a reference, and IgG serves as a negative control for the qRT‐PCR reaction. The results are analyzed using the 2^−ΔΔCt^ method.

### 5.15 ChIP‐seq Library Construction, Preprocessing, and Data Analysis

Ovaries were obtained from Control and PAE neonates at GD18. Approximately 50 neonatal ovaries were pooled per biological replicate to obtain sufficient chromatin. Tissues were cross‐linked with 1% formaldehyde to form covalent protein‐DNA complexes, and the reaction was quenched with glycine. Cells were lysed to release cytoplasmic contents, and nuclei were collected. Nuclear chromatin was extracted and fragmented via sonication to generate DNA fragments ranging from 200 to 1000 bp. Immunoprecipitation was performed using specific antibodies against HDAC1 and H3K27ac, with normal IgG serving as a negative control. The efficiency of immunoprecipitation was verified by Western Blot analysis. Following incubation, antibody‐chromatin complexes were washed, eluted, and subjected to reverse cross‐linking. DNA was subsequently purified for library preparation.

Chromatin immunoprecipitation sequencing libraries were sequenced on an Illumina platform using a Paired‐End 150 bp (PE‐150) protocol. Raw sequencing reads were subjected to quality control using FastP to remove low‐quality bases and trim adapter sequences [41]. Cleaned reads were aligned to the mouse reference genome (UCSC mm10 p6) using Bowtie2 [42]. Post‐alignment processing included the removal of PCR duplicates and filtering of unmapped reads to generate high‐confidence alignment files (BAM) for downstream analysis. To identify genomic regions enriched for HDAC1 and H3K27ac, peak calling was performed using Epic2 [43], an algorithm optimized for the detection of broad chromatin domains. Peaks were called for each immunoprecipitated sample against its corresponding Input control using default parameters. Genomic annotation of identified peaks (promoters, exons, introns, and intergenic regions) was conducted using the ChIPseeker R package with a defined promoter transcription start site (TSS) region of 2 ± kb. Differential binding affinity between the PAE and Control groups was assessed using the csaw R package, which utilizes a window‐based counting approach to detect changes in chromatin occupancy independent of pre‐defined peaks. Reads were counted in non‐overlapping windows, and background enrichment was filtered based on global abundance. The edgeR statistical framework was employed within csaw to calculate normalization factors (TMM method) and estimate tagwise dispersion [44]. Differentially bound regions were identified using a Generalized Linear Model (GLM). For data visualization, coverage tracks (BigWig files) were generated using deepTools (bamCoverage) with RPKM normalization. Heatmaps and profile plots illustrating signal enrichment around the TSS were generated using computeMatrix and plotHeatmap (deepTools) [45]. Genomic browser tracks were visualized using IGV. To characterize the relationship between HDAC1 activity and histone acetylation, we utilized publicly available ChIP‐seq datasets from the Gene Expression Omnibus (GEO) under accession number GSE193085. This dataset comprises H3K27ac profiles in Mouse Embryonic Stem Cells (mESCs) treated with the HDAC inhibitor Sodium Butyrate (NaB) versus untreated controls. Raw sequencing data (FASTQ format) were downloaded using the SRA Toolkit. To ensure direct comparability with our generated datasets, the raw public data were processed using the identical bioinformatics pipeline described above. Briefly, reads were quality‐trimmed using FastP, aligned to the mouse reference genome (UCSC mm10) using Bowtie2, and filtered to remove PCR duplicates. BigWig coverage tracks were generated using deepTools with RPKM normalization to facilitate visual comparison of H3K27ac signal intensity at the Usp9x promoter between NaB‐treated cells and our PAE neonatal ovary samples.

### 5.16 Measurement of Total HDAC and HDAC1 Enzymatic Activity

Total HDAC activity was measured using the Mouse Histone Deacetylase (HDAC) ELISA Kit (BY‐EM220881, BYabscience, Nanjing, China). HDAC1‐specific activity was determined using the Mouse Histone Deacetylase 1 (HDAC1) Quantitative Detection Kit (BY‐EM227946, BYabscience, Nanjing, China). Ovaries were obtained from Control and PAE neonates at GD18. Approximately 30 neonatal ovaries were pooled per biological replicate (10mg/sample). Tissue samples were homogenized in PBS (0.01 m, pH 7.4) containing protease inhibitors, and nuclear proteins were extracted. Samples, standards, and controls were added to microplate wells pre‐coated with anti‐HDAC or anti‐HDAC1 capture antibodies. After incubation at 37°C for 60 min, wells were washed five times to remove unbound components. A horseradish peroxidase (HRP)‐conjugated detection antibody specific for HDAC or HDAC1 was then added and incubated for 60 min at 37°C. Following a second wash step, substrate solutions A and B (1:1 ratio) were added, and the reaction was allowed to proceed for 15 min at 37°C in the dark. The reaction was stopped with 2 m sulfuric acid, and the optical density (OD) was measured at 450 nm using a microplate reader.

Enzymatic activity was determined by interpolation from a standard curve generated using recombinant mouse HDAC (range: 12.5–400 pmol/L) or HDAC1 (range: 0.625–20 ng/mL) standards. Results were normalized to total protein concentration and expressed as relative activity compared to controls.

### 5.17 Statistical Analysis

Data collection and analysis were performed using Microsoft Excel (Microsoft, Redmond, WA, USA) and GraphPad Prism 9.0 (GraphPad Software, La Jolla, CA, USA). All quantitative data are presented as the mean ± standard error of the mean (S.E.M.). Prior to significance testing, data distribution was assessed for normality using the Shapiro–Wilk test. For comparisons between two groups, Student's two‐tailed *t*‐test was used. For comparisons involving three or more groups (e.g., dose‐response experiments), one‐way analysis of variance (ANOVA) was performed, followed by Tukey's post hoc test for multiple comparisons. For fetal weights, the mean weight of each litter was treated as the experimental unit. The precise sample size (n) for each experiment is explicitly stated in the corresponding figure legends. A *p*‐value of < 0.05 was considered statistically significant (^*^ *p* < 0.05, ^**^ *p* < 0.01).

### 5.18 Ethical Statement

Animal experiments were conducted at the Center for Animal Experiments, Wuhan University, accredited by AAALAC International. All procedures were approved by the Experimental Animal Welfare Ethics Committee of Zhongnan Hospital, Wuhan University (permit number: ZN2022228), following NIH regulations and ARRIVE guidelines. It is confirmed that this study meets the ethical guidelines outlined in this journal's Author Guidelines, including adherence to the legal requirements of the study country.

## Author Contributions

H.W. led the conceptualization and resources and contributed to funding acquisition. Y.L. contributed equally to the conceptualization and led data curation, investigation, methodology, visualization, original draft preparation, and manuscript review and editing. C.G. contributed equally to funding acquisition, investigation, supervision, validation, and manuscript review and editing. W.Y.Y. contributed equally to data curation, investigation, visualization, and manuscript review and editing. H.F. contributed to data curation and methodology. T.W. contributed to data curation, methodology, and original draft preparation. L.C. contributed to formal analysis, investigation, and supervision. Q.X. and S.P. contributed to data curation and methodology. M.W. and H.C. contributed to methodology and manuscript review and editing. Y.Z. led funding acquisition and contributed equally to project administration and supervision, and to manuscript review and editing.

## Conflicts of Interest

The authors declare no conflicts of interest.

## Supporting information




**Supporting File 1**: advs73748‐sup‐0001‐SuppMat.docx.

**Supporting File 2**: advs73748‐sup‐0002‐Supplementary Table 1.xls.

**Supporting File 3**: advs73748‐sup‐0003‐Supplementary Table 2.xlsx.

## Data Availability

The data that support the findings of this study are available from the corresponding author upon reasonable request.
