# Supplementary material for: USP9X as a Candidate Mediator of Prenatal Aspirin‐Induced Ovarian Reserve Reduction in Offspring Mice
Source: Adv Sci (Weinh). 2026 Jan 12;13(16):e07679. doi: 10.1002/advs.202507679 (PMC13042442; doi:10.1002/advs.202507679)
Supplement: Supplementary file 1 — Supporting File 1: advs73748‐sup‐0001‐SuppMat.docx. [file ADVS-13-e07679-s002.docx]

Supporting Information

**USP9X as a Candidate Mediator of Prenatal Aspirin-Induced Ovarian Reserve Reduction in Offspring Mice**

*Yating Li^1, 2, #^, Caiyun Ge^3, 4, #^, Wai Yen Yim^5^, Hui Feng^1^, Tiancheng Wu^1^, Lu Chen^2^, Qiaohua Xiong^1^, Shumin Pan^1^, Mei Wang^4^, Huijun Chen^1, 3^, Yuanzhen Zhang^1, 3, 4, 6, 7, *^, Hui Wang^1, 2, 3, *^*

**
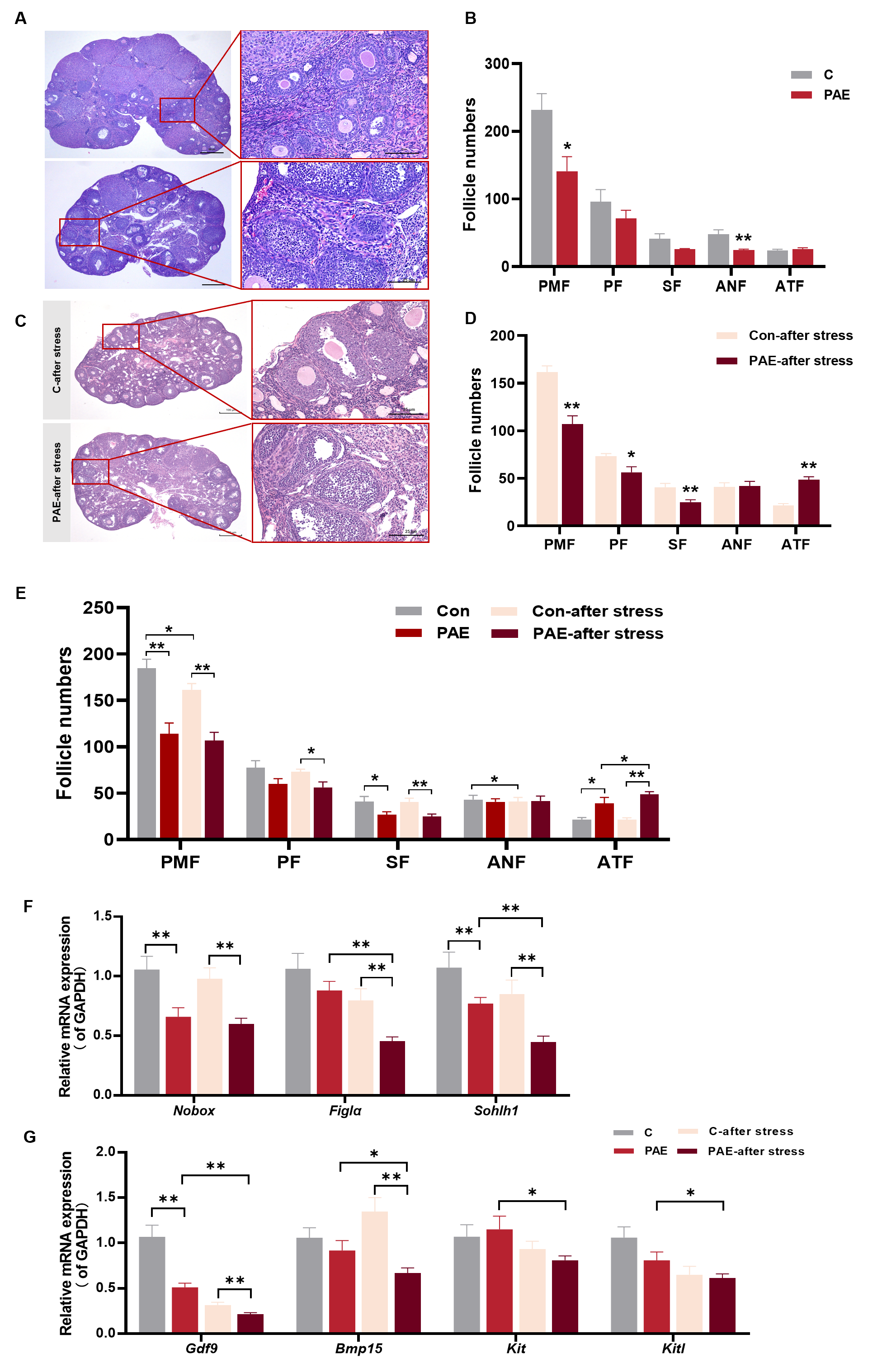
**

**Figure S1. Effects of PAE in PW6 and PW12 after stress offspring’s ovaries. (A)** Ovarian morphology by H&E staining in PW6 offspring, n=5; **(B)** Number of ovarian follicles at each stage in PW6 offspring; **(C)** Ovarian morphology by H&E staining in PW12 offspring after stress, n=5; **(D)** Number of ovarian follicles at each stage in PW12-after stress offspring; **(E)** Number of ovarian follicles at each stage in PW12 and PW12-after stress offspring; **(F)** The relative mRNA expression of *Nobox, Figlα, and Sohlh1,* n=10; **(G)** The relative mRNA expression of *Gdf9, Bmp15, Kit*, and *Kitl,* n=10; Mean ± S.E.M. ^*^*P<*0.05, ^**^*P<*0.01 *vs*. control. PAE, prenatal aspirin exposure; PW, postnatal week; Nobox, NOBOX oogenesis homeobox; *Figlα*, factor in the germline alpha; *Sohlh1*, spermatogenesis and oogenesis specific basic helix-loop-helix 1; *Gdf9*, growth differentiation factor 9; *Bmp15*, bone morphogenetic protein 15; *Kit*, receptor tyrosine kinase; *Kitl*, kit ligand; PMF, primordial follicle; PF, primary follicle; SF, secondary follicle; ANF, antral follicle; ATF, atretic follicles.

**
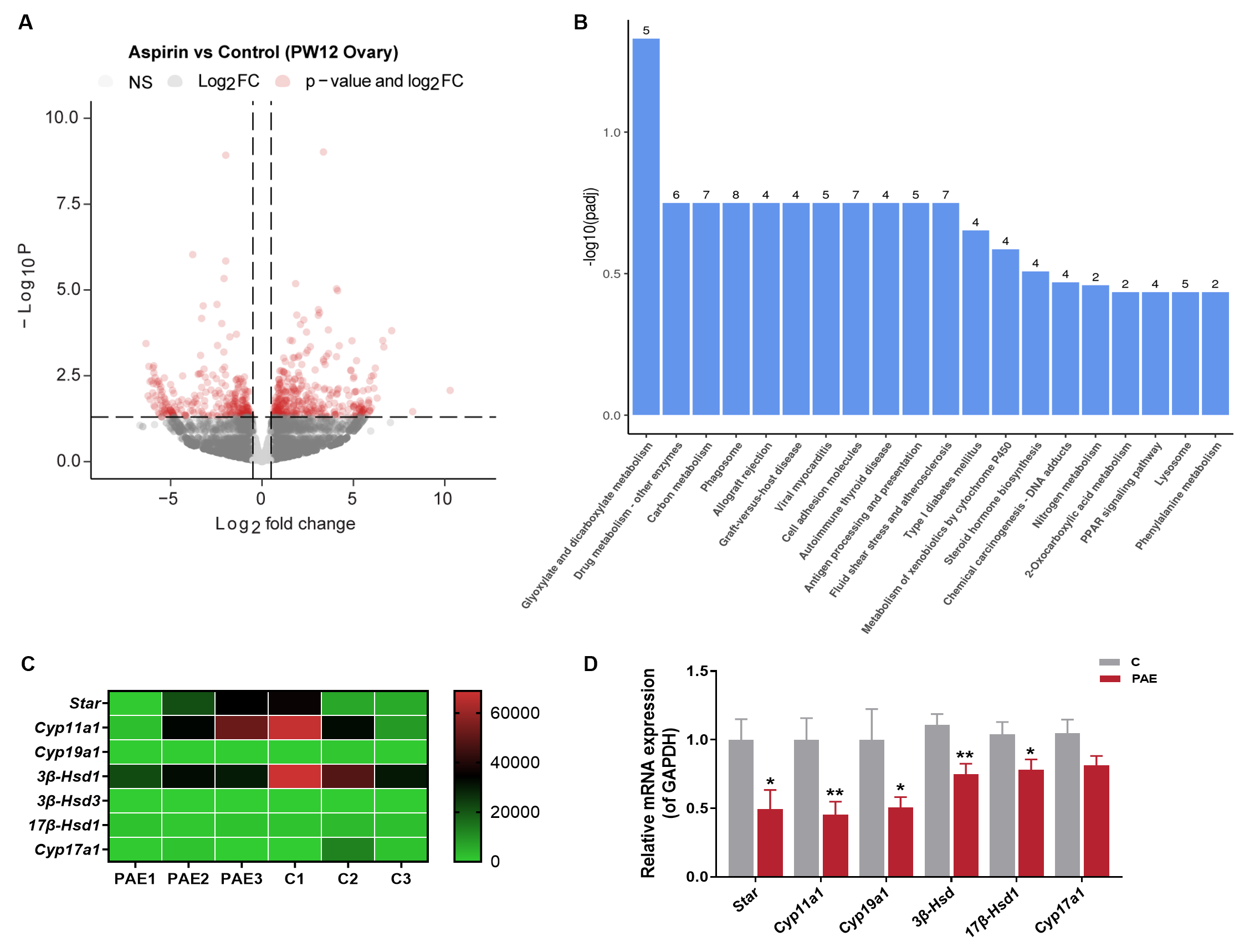
**

**Figure S2. Effects of PAE on ovarian gene expression and histological changes in PW12 offspring. (A)** Differential gene analysis of RNA sequencing in the ovaries of PW12 offspring; **(B)** KEGG enrichment analysis of RNA sequencing results; **(C)** Heatmap of genes related to steroid synthesis pathway changes from Reactome enrichment analysis; **(D)** The relative mRNA expression of steroid genesis-related genes *Star, Cyp11a1, Cyp19a1, 3β-Hsd1, 17β-Hsd1,* and *Cyp17a1*, n=10; Mean ± S.E.M. ^*^*P*<0.05, ^**^*P*<0.01 vs. control. PAE, prenatal aspirin exposure; PW, postnatal week; KEGG, Kyoto Encyclopedia of Genes and Genomes; *Star,* steroidogenic acute regulatory protein; *Cyp11a1*, cytochrome P450 family 11 subfamily A member 1; *Cyp19a1*, cytochrome P450 family 19 subfamily A member 1; *3β-Hsd1*, hydroxysteroid 3-beta dehydrogenase 1; *17β-Hsd1,* hydroxysteroid 17-beta dehydrogenase 1; *Cyp17a1*; cytochrome P450 family 17;


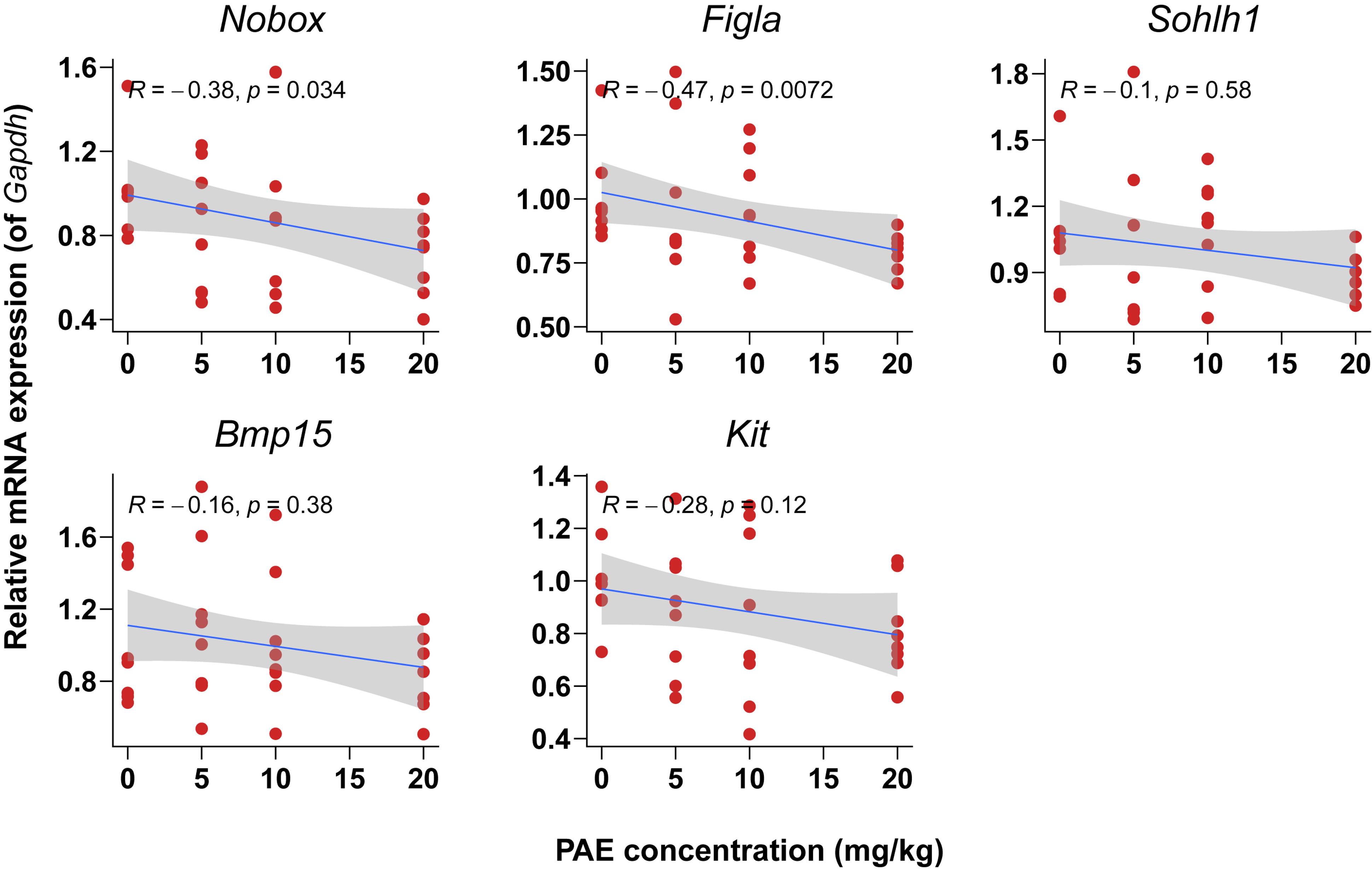


**Figure S3. Correlation of Target Gene Expression of Offspring Ovary with PAE Dosage.** Scatterplots showing the correlation between aspirin dosage (X-axis) and relative gene expression (Y-axis) for: *Nobox, Figla, Sohlh1, Bmp15,* and *Kit*. The line of best fit from a linear regression is shown. The Spearman rank correlation coefficient (R) and p-value are displayed for each gene. Statistical significance is noted as *P* < 0.05, **P* < 0.01, and ***P* < 0.005.


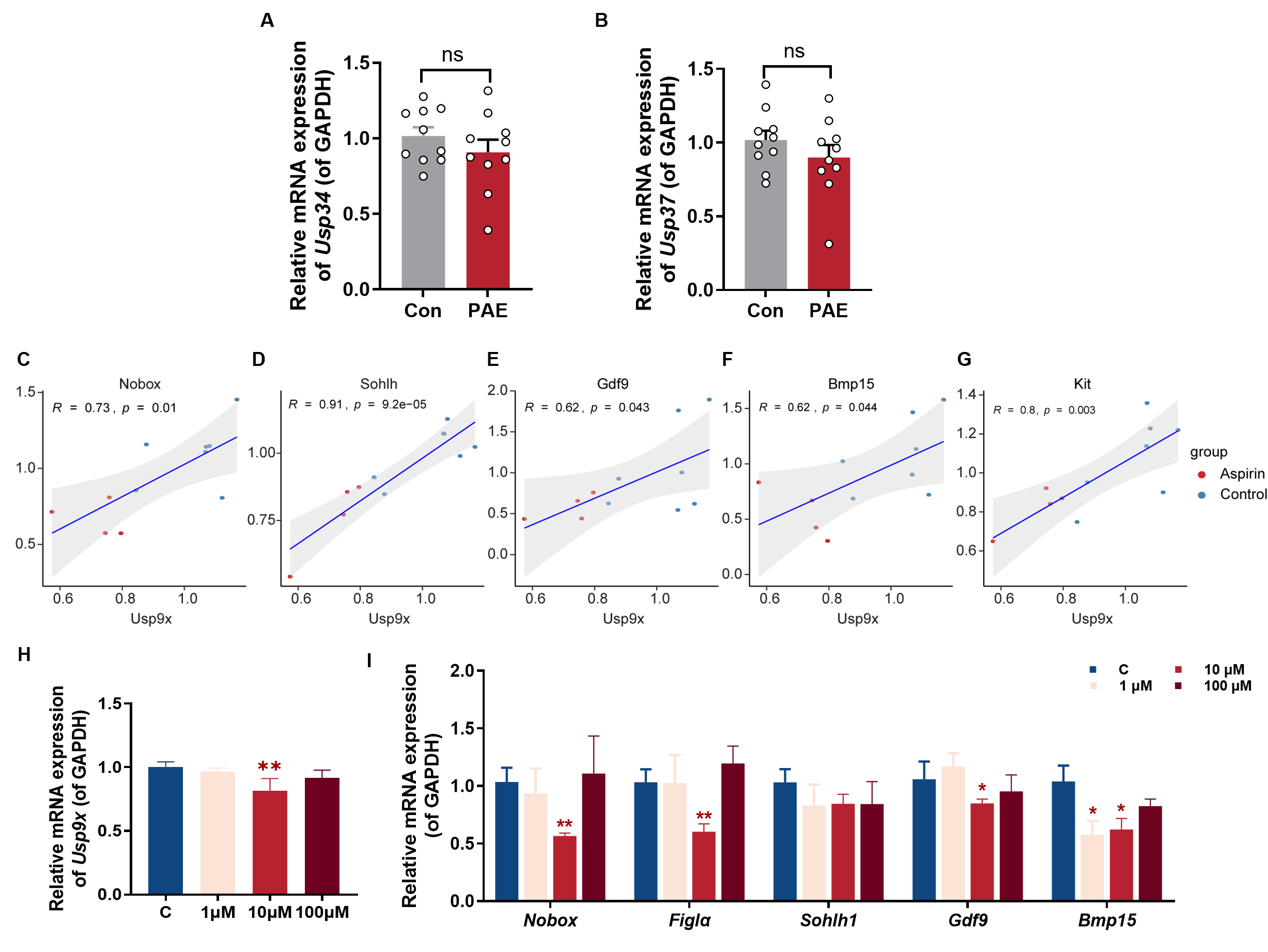


**Figure S4. Impact and correlation of PAE on gene expression related to ovarian development.** **(A)** The relative mRNA expression of *Usp34* in the ovaries of PAE offspring at GD18, n=10; **(B)** The relative mRNA expression of *Usp37* in the ovaries of PAE offspring at GD18, n=10; **(C-G)** Correlation analysis of the relative mRNA expression of *Usp9x* with *Nobox, Sohlh1, Gdf9, Bmp15*, and *Kit* in PAE fetal ovaries; **(H)** The relative mRNA expression of *Usp9x* in fetal ovaries cultured in vitro under varying concentrations of aspirin (1, 10, 100 μM), n=10; **(I)** The relative mRNA expression of *Nobox, Figlα, Sohlh1, Gdf9*, and *Bmp15* in fetal ovaries cultured *in vitro*, n=10; Mean ± S.E.M. ^*^*P<*0.05, ^**^*P<*0.01 *vs*. control. PAE, prenatal aspirin exposure; *Usp9x*, ubiquitin specific peptidase 9, X-linked; *Nobox*, NOBOX oogenesis homeobox; *Figlα*, factor in the germline alpha; *Sohlh1*, spermatogenesis and oogenesis specific basic helix-loop-helix 1; *Gdf9*, growth differentiation factor 9; *Bmp15*, bone morphogenetic protein 15.


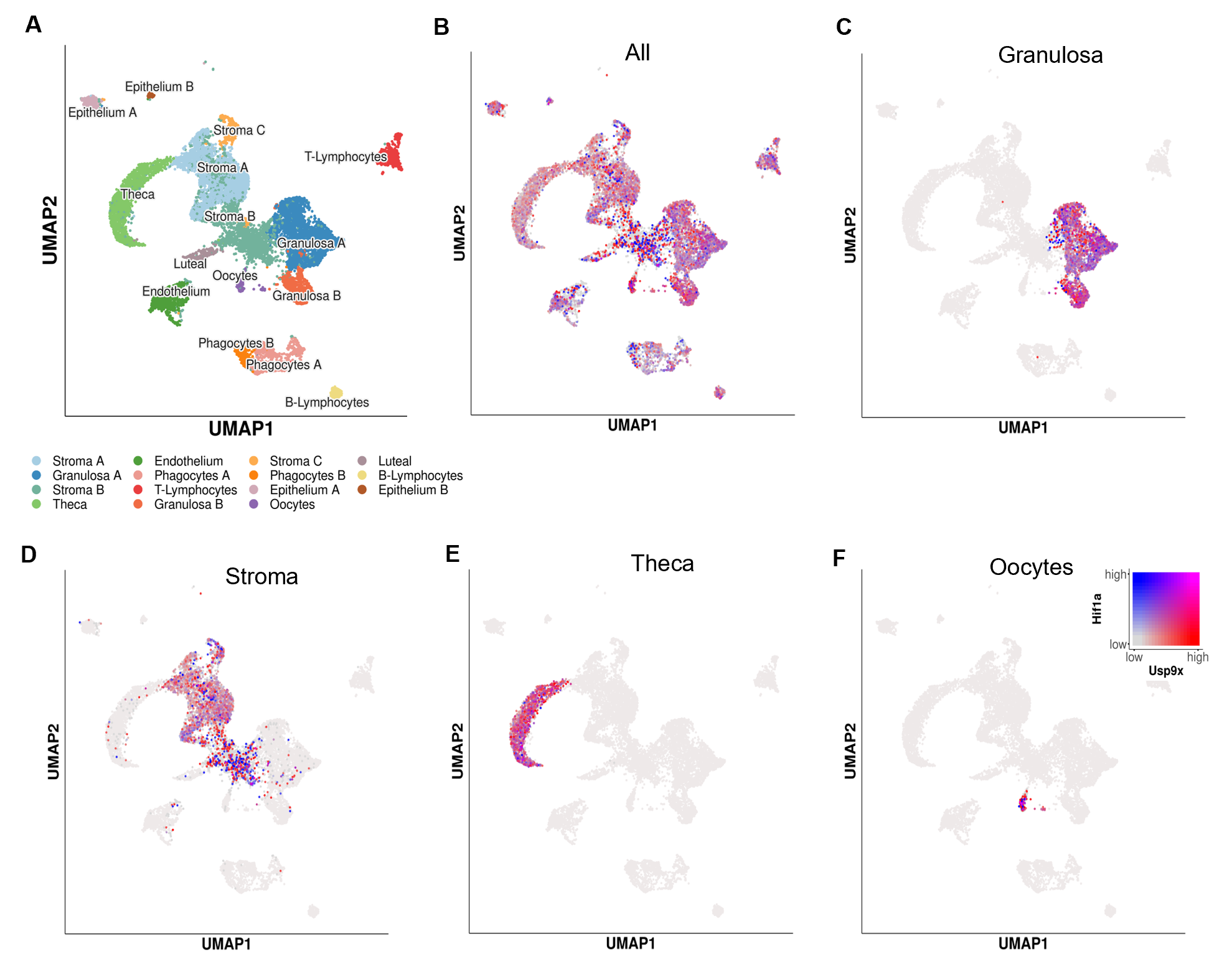


**Figure S5. Analysis of USP9X, HIF1α, and their co-expression across ovarian cell subpopulations using single-cell RNA sequencing of a GEO database.** **(A)** Cell clustering analysis of ovarian tissue; **(B)** Co-expression of USP9X and HIF1α across all ovarian cells; **(C)** Co-expression of USP9X and HIF1α in granulosa cells; **(D)** Co-expression of USP9X and HIF1α in stromal cells; **(E)** Co-expression of USP9X and HIF1α in theca cells; **(F)** Co-expression of USP9X and HIF1α in oocytes. The database is derived from the NCBI Gene Expression Omnibus (GEO), accession number GSE232309. USP9X, ubiquitin specific peptidase 9, X-linked; HIF1α, hypoxia inducible factor-1α.

|  |  |
| --- | --- |


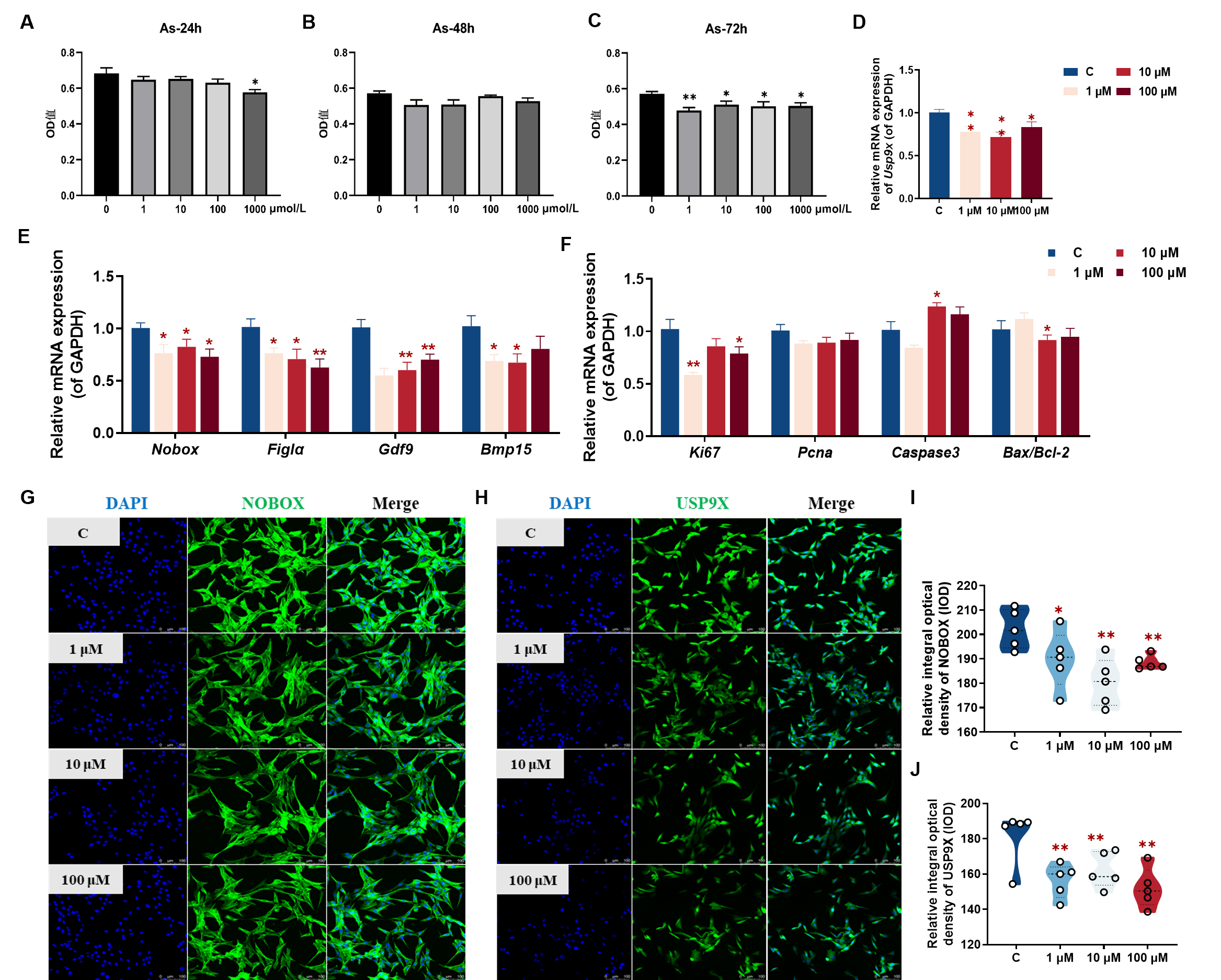


**Figure S6. Effects of different concentrations and time points of aspirin treatment on NIH3T3 cell viability and gene expression. (A-C)** Cell viability assay at different time points (24h, 48h, 72h) and concentrations ((1 μM, 10 μM, 100 μM, 100 μM) of aspirin; **(D)** The relative mRNA expression of *Usp9x*, n=6; **(E)** The relative mRNA expression of *Nobox, Figlα, Gdf9*, and *Bmp15*, n=6; **(F)** The relative mRNA expression of *Ki67, Pcna, Caspase3, Bax,* and *Bcl-2*, n=6; **(G, H)** Immunofluorescence staining of NOBOX and USP9X (400×), n=5; **(I, J)** The relative mean fluorescence intensity of NOBOX and USP9X. Mean ± S.E.M. ^*^*P<*0.05, ^**^*P<*0.01 *vs*. control. PAE, prenatal aspirin exposure; USP9X, ubiquitin specific peptidase 9, X-linked; NOBOX, NOBOX oogenesis homeobox; *Figlα*, factor in the germline alpha; *Gdf9*, growth differentiation factor 9; *Bmp15*, bone morphogenetic protein 15; *Ki67*, antigen identified by monoclonal antibody Ki-67; *Pcna*, proliferating cell nuclear antigen; *Caspase3*, cysteine-dependent aspartate-specific proteases 3; *Bax*, Bcl-2-associated X protein; *Bcl-2*, Bcl-2-associated X protein.


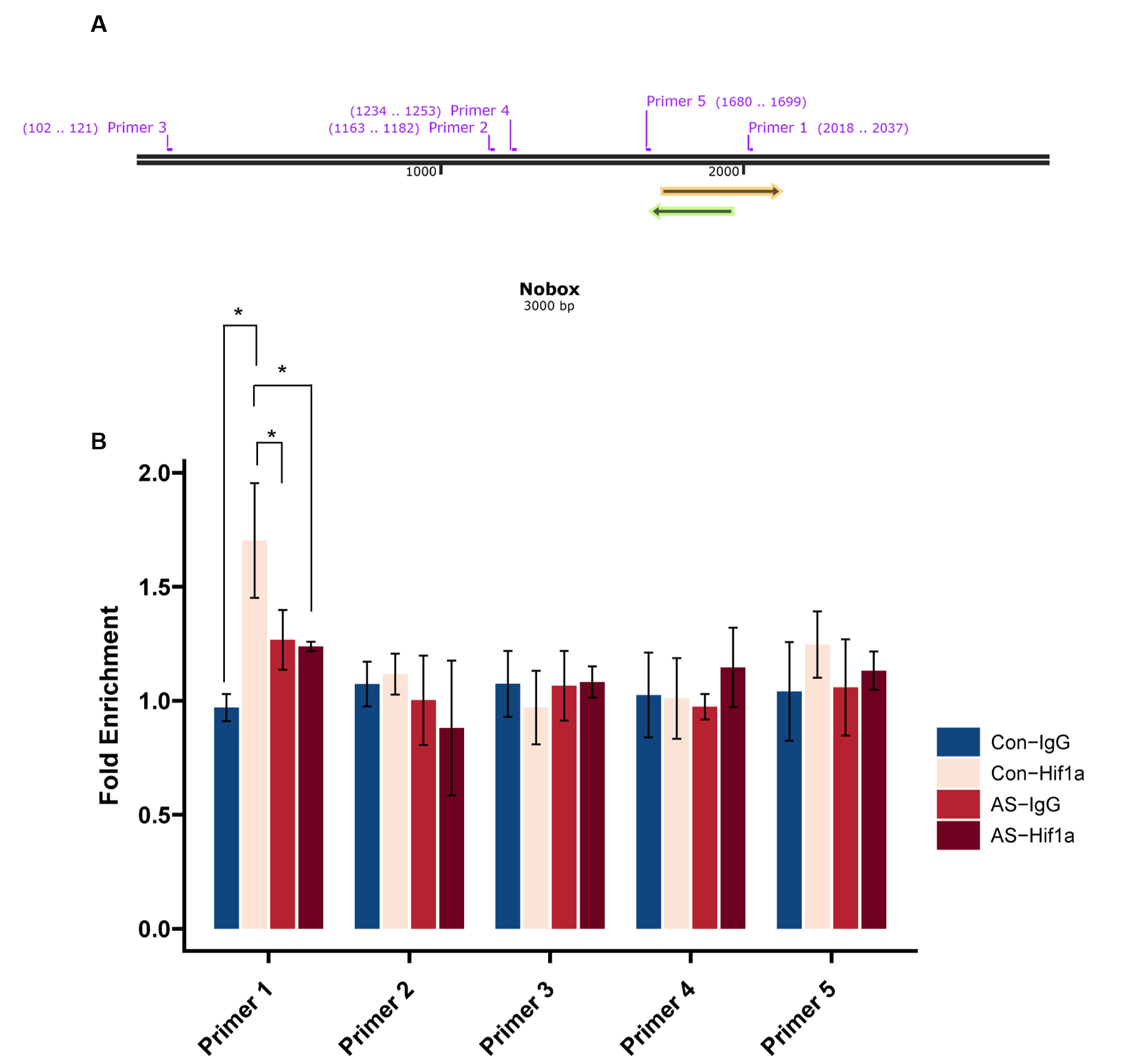


**Figure S7. PCR primer design and ChIP-qPCR validation of HIF1α enrichment at the *Nobox* promoter in NIH3T3 cell line**. **(A)** Schematic representation of the *Nobox* promoter region generated using SnapGene. The diagram indicates the precise genomic coordinates and relative positions of the five primer sets (Primers 1-5) designed for ChIP-qPCR analysis. **(B)** ChIP-qPCR quantification of HIF1α enrichment across the *Nobox* promoter regions targeted by Primers 1–5. Bar graphs display fold enrichment relative to the IgG negative control in Control (Con). Data are presented as mean ± SEM.


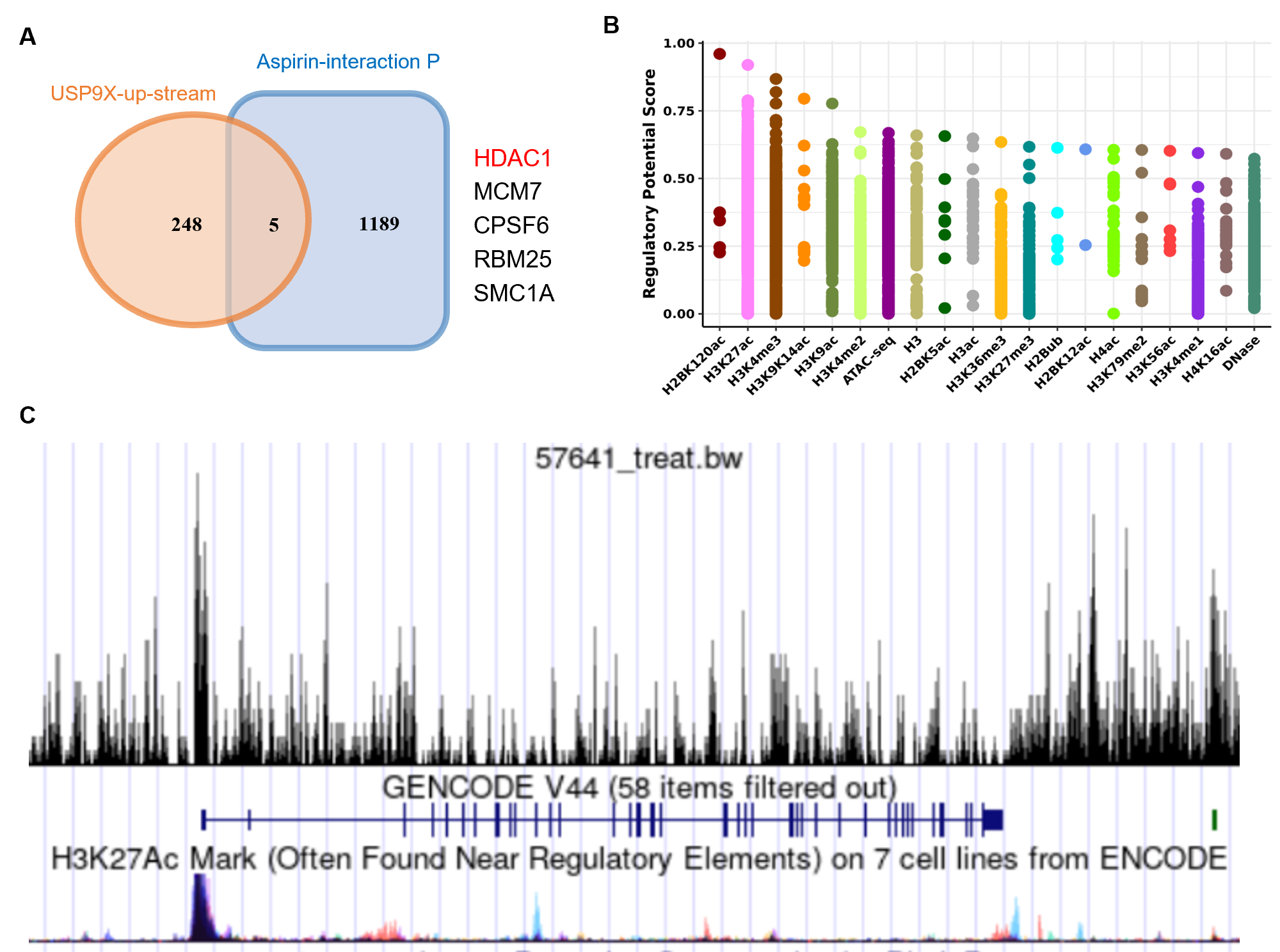


**Figure S8. Analysis of USP9X promoter binding proteins. (A)** Venn diagram of the intersection between aspirin-binding target proteins and USP9X promoter binding proteins (generated using Draw Venn Diagram); **(B)** Prediction and ranking of epigenetic modification sites in the Usp9x gene from Encode and UCSC websites; **(C)** Enrichment of HDAC1 at the *Usp9x* gene promoter region as analyzed by the Cistrome database; Mean ± S.E.M. ^*^*P<*0.05, ^**^*P<*0.01 *vs*. control. USP9X, ubiquitin specific peptidase 9, X-linked; HDAC1, histone deacetylase 1.


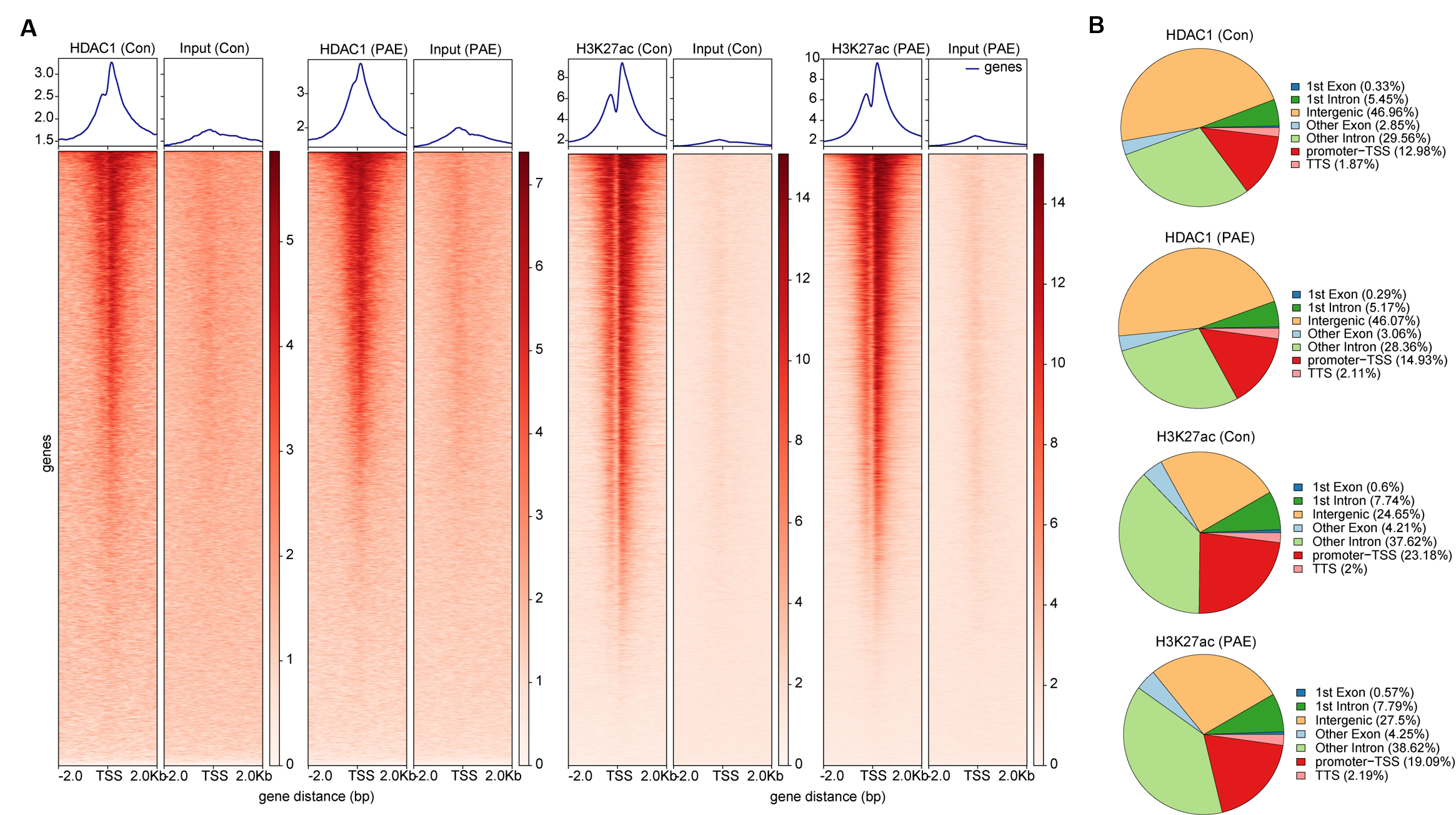


**Figure S9.** **Quality control and genomic distribution of HDAC1 and H3K27ac ChIP-seq datasets.** (A) Heatmap visualization of ChIP-seq signal enrichment centered on the Transcription Start Site (TSS) of annotated genes. The panel displays read density for HDAC1 and H3K27ac in Control and PAE conditions, alongside their respective input controls. Rows represent individual genomic regions sorted by signal intensity, covering a window of [insert window size, e.g., ±3 kb] around the TSS. (B) Genomic annotation of ChIP-seq peaks. Pie charts illustrate the distribution of HDAC1 and H3K27ac binding sites across defined genomic features, including promoters (TSS), exons (1st and other), introns (1st and other), and intergenic regions.


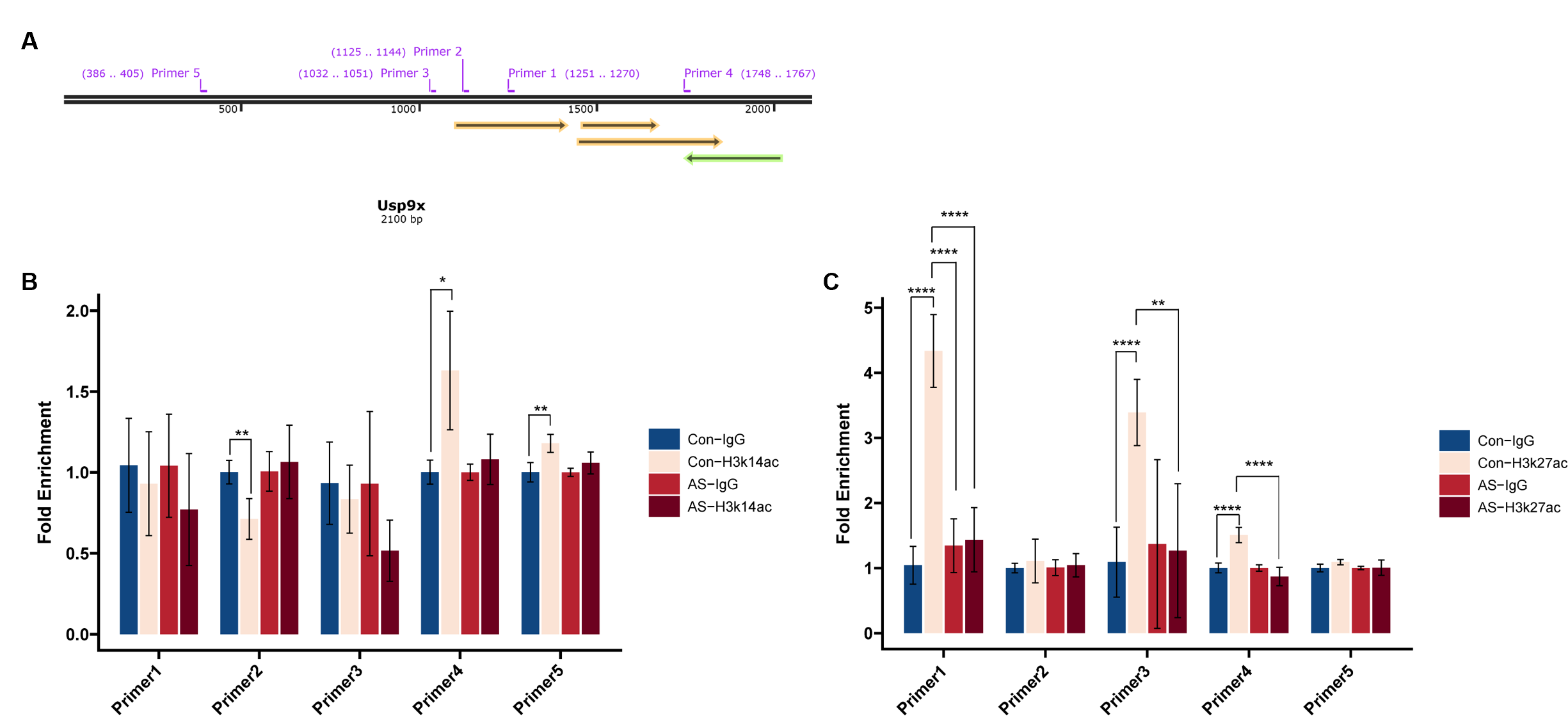


**Figure S10. PCR primer design and ChIP-qPCR validation of H3K14ac and H3K27ac enrichment at the *Usp9x* promoter in NIH3T3 cell line**. **(A)** Schematic representation of the *Usp9x* promoter region generated using SnapGene. The diagram indicates the precise genomic coordinates and relative positions of the five primer sets (Primers 1-5) designed for ChIP-qPCR analysis. **(B-C)** ChIP-qPCR quantification of (B) H3K14ac and (C) H3K27ac enrichment across the *Usp9x* promoter regions targeted by Primers 1-5. Bar graphs display fold enrichment relative to the IgG negative control in Control (Con). Data are presented as mean ± SEM.

**
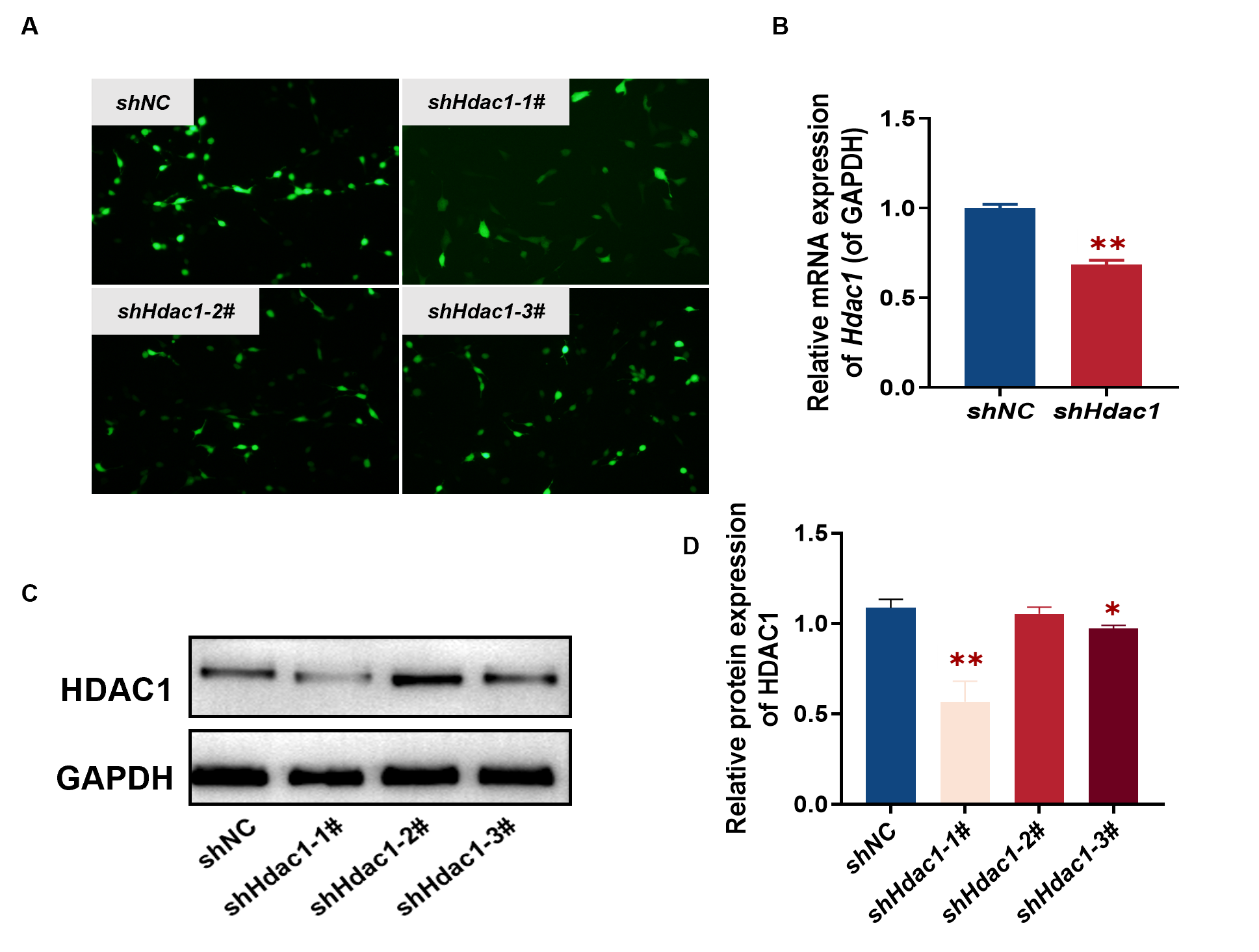
**

**Figure S11. HDAC1 knockdown experiments. (A)** GFP fluorescence labeling of shHdac1-1#, shHdac1-2#, shHdac1-3#, and shNC transfected into NIH3T3 cells; **(B)** The relative mRNA expression of *Hdac1* in NIH3T3 cells after transfection with shHdac1-1#; **(C)** WB analysis of HDAC1 protein levels following transfection with shHdac1-1#, shHdac1-2#, and shHdac1-3#; (D) The relative protein expression of HDAC1; Mean ± S.E.M. ^*^*P<*0.05, ^**^*P<*0.01 *vs*. control. HDAC1, histone deacetylase 1.
